# Janus-Structured Micro/Nanomotors: Self-Propelled Mechanisms and Biomedical Applications

**DOI:** 10.34133/bmr.0155

**Published:** 2025-04-05

**Authors:** Haoyan Cheng, Beng Ma, Anqi Ji, Haonan Yao, Pan Chen, Wenyang Zhai, Shegan Gao, Linlin Shi, Hao Hu

**Affiliations:** ^1^ School of Materials Science and Engineering, The First Affiliated Hospital of Henan University of Science and Technology, Henan University of Science and Technology, Luoyang 471023, P. R. China.; ^2^ Henan Key Laboratory of Microbiome and Esophageal Cancer Prevention and Treatment, Henan Key Laboratory of Cancer Epigenetics, The First Affiliated Hospital of Henan University of Science and Technology, Luoyang 471023, P. R. China.

## Abstract

Self-propelled micro/nanomotors (MNMs), which can convert other energy into mechanical motion, have attracted considerable attention due to their potential applications in diverse fields. Due to the asymmetric structures and 2 or more chemically discrepant composites constructed in the Janus nanoparticles, asymmetrical forces can be created in the physical environment. Thus, MNMs with Janus structures have been widely studied for revealing possible driving mechanisms. This tutorial review covers the most representative examples of Janus-structured MNMs developed so far, which are self-propelled by different mechanisms. We focus on Janus MNMs that exhibit self-propelled motion in liquid environments and their potential applications in biomedicine, including drug delivery, cancer therapy, bioimaging, and biosensing. The driving mechanisms and challenges associated with constructing asymmetric fields are deeply discussed, along with future opportunities for these versatile and promising MNMs. This review provides an overview of the rapidly evolving field of MNMs and their potential applications, serving as a valuable resource for researchers and others interested in this field.

## Introduction

Micro/nanomotors (MNMs) are micro/nanoscaled devices capable of converting different forms of energy into mechanical energy and exhibiting self-propelled motion in liquid environments [1]. Due to their unique autonomous motion and the ability to load, transport, and release cargoes in a liquid medium, self-propelled MNMs have gained tremendous attention in multiple areas, including cancer diagnosis and therapy, biosensing, environmental remediation, and advanced nanomanufacturing [2]. Their autonomous mobility effectively penetrates biological barriers [3], such as dense extracellular matrix [4,5], blood–brain barrier [6], or blood–tumor barrier, presenting great potential for targeted drug delivery and deep tumor penetration [7]. Based on their driving force source, existing MNMs can be classified into chemical-driven MNMs (H_2_O_2_, Urea, H_2_O, N_2_H_4_, etc.) and external field-driven MNMs (ultrasonic field, magnetic field, optical field, electric field) (Fig. S1). The motion mechanisms of MNMs include bubble propulsion [8], self-diffusiophoresis [9], and self-electrophoresis [10]. Among these, bubble recoil motion depends on the continuous movement of the motor driven by the momentum change as bubbles are expelled from it [11]. Self-phoresis-driven MNMs operate through the asymmetric release of products or heat, creating local gradients in electric potential, concentration, temperature, or surface tension that induce fluid flow and propel the motor [12]. By constructing asymmetric fields, the static balance of MNMs is broken, with most MNMs requiring unique structures. Janus nanoparticles (JNPs) with 2 distinct faces have become a primary focus in the field of MNMs because they integrate diverse components into a single structure to obtain constant gradients. This breaks the traditional symmetry and provides increased versatility [13]. A common method for creating JNPs involves distributing them as a monolayer on a substrate and then using physical or chemical vapor deposition (PVD/CVD) to coat one hemisphere, forming the Janus structure [14]. However, these techniques are expensive and require stringent manufacturing conditions [13], making them less suitable for fabricating small JNPs prone to aggregation. Alternatives like chemical synthesis and template-assisted methods can guide particle distribution and functionalization, allowing for customizable morphology and size in Janus nanomotors (JNs) [15]. These diverse fabrication methods enable researchers to select the most appropriate approach based on specific application needs, such as precision, cost, and material properties. Given the rapid progress in self-propelled MNM research, it is necessary to provide timely updates on Janus-structured self-propelled MNMs. This review covers the development, properties, and classification of Janus particles (JPs) with controlled shapes and descriptions of their driving mechanism [16]. It also highlights their remarkable application in drug delivery, cancer therapy, bioimaging, and biosensing. Furthermore, we discuss present challenges and opportunities, offering insight into the future development of Janus MNMs and pushing them closer to application. It is hoped that this article will provide a comprehensive overview of the rapidly evolving field of MNMs for the benefit of researchers and those interested in MNM applications.

## Chemically Powered Janus MNMs

The motion of nanoscale objects presents considerable challenges in multidisciplinary nanotechnology due to Brownian diffusion. One solution to achieving continuous motion in these regimes is externally actuated MNMs, which can power chemical energy, electric field, magnetic field, or ultrasound field. Among them, chemically powered MNMs based on chemical reactions and energy conversion show great potential for applications [17]. To design chemically driven MNMs, it is crucial to explore chemical fuels that can disrupt the static force balance of the device through a particular chemical reaction. Janus MNMs can be driven by the decomposition of fuel substances like aqueous peroxide (H_2_O_2_) [18–21], hydrazine (N_2_H_4_), water (H_2_O), and other chemical fuels [22], which generate propulsion via bubble recoil mechanisms or phoresis [23].

### H_2_O_2_-powered MNMs

H_2_O_2_ is commonly used as a fuel in chemically driven MNMs, where the decomposition of H_2_O_2_ into H_2_O and O_2_ with the help of catalysts results in the actuation of MNMs through bubble recoil or phoresis mechanisms. The catalysts used in H_2_O_2_-powered MNMs are diverse, including metallic catalysts (e.g., Pt) [24], transition metal oxides (e.g., MnO_2_ [25] and Fe_3_O_4_ [26]), and biocatalysts such as enzymes [27–29]. Among them, Pt is the most widely used catalyst for H_2_O_2_-powered MNMs due to its high efficiency and stability. For example, Paxton et al*.* [30] developed nanorod motors composed of Pt and Au segments. In an H_2_O_2_ solution, these segments serve as electrodes in a galvanic cell, with H_2_O_2_ undergoing oxidation at the Pt end and reduction at the Au end (Eqs. 1 and 2). This reaction asymmetry leads to a higher proton concentration near the Pt side, creating a self-induced electric field that propels the negatively charged nanorods toward the Pt end via self-electrophoresis (Fig. S2A).

$$\mathrm{Pt}\;:H_{2}O_{2}\to O_{2}+{\text{2e}}^{-}+{\text{2H}}^{+}$$ (1)

$$\mathrm{Au}\;:H_{2}O_{2}+{\text{2H}}^{+}+{\text{2e}}^{-}\to {\text{2H}}_{2}O$$ (2)

Ma et al. [31] reported the fabrication of reversed Janus MNMs with an internal chemical engine (Fig. S2B). Pt nanoparticles are embedded within the interior of the hollow SiO_2_. When the Janus MNM is suspended in an aqueous H_2_O_2_ solution, it triggers the decomposition of H_2_O_2_, driving the motion of the Janus MNM (Fig. S2C to E). The propulsion force of these MNMs depends on factors such as the concentration gradient of the product (Fig. S2F and G). The catalytic activity of traditional Pt-based MNMs is often insufficient to promote self-propulsion in relatively dilute H_2_O_2_ solutions. However, recent research has discovered that surface catalytic performance can be regulated through heteroatom doping and alloying strategies in Pt-based MNMs, thus facilitating the mechanical movement of nanomotors. Xue and colleagues [32] synthesized JNs by depositing an ultrathin metal layer (containing Pt, Pd, and Mo) on carbonaceous nanospheres, successfully constructing a ternary alloy-based engine as shown in Fig. S2H. The morphology and corresponding element mapping were depicted in Fig. S2I. Density functional theory calculations suggested that the alloyed engine showed higher H_2_O_2_ absorptivity and catalytic efficiency than the standard Pt-based engine, resulting in more effective degradation to O_2_ and H_2_O due to optimized free energy of each transition state (Fig. S2J and K).

**Fig. 1.**
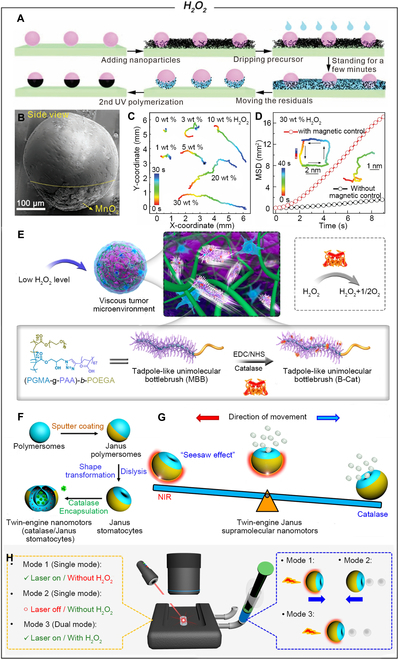
(A) Fabrication of hydrogel-based JMs capped with different coverages of functional nanoparticles. (B) Scanning electron microscopy (SEM) images of hydrogel-based micromotors covered with MnO_2_ nanoparticles. (C) Trajectories of MnO_2_ JMs in various concentrations of H_2_O_2_. (D) Motion characterization of multifunctional MnO_2_ and Fe_3_O_4_ JMs. Reproduced from [37] with permission. Copyright 2020 The Authors. Published by Wiley-VCH Verlag GmbH & Co. KGaA, Weinheim. (E) Schematic representation of tadpole-like unimolecular bottlebrush nanomotor. Reproduced with permission from [39]. Copyright 2019 American Chemical Society. (F) Schematic representation of the steps involved in preparing twin-engine CAT/Janus stomatocytes. (G) Seesaw effect of the twin-engine Janus supramolecular nanomotors. (H) Schematic depicting the characterization of motion behavior. Reproduced with permission from [40]. Copyright 2022 The Authors. Published by American Chemical Society.

Although Pt-based MNMs exhibit excellent performance in H_2_O_2_ decomposition, their relatively high costs limit their scale-up applications. In contrast, Mn-based materials with low prices and high catalytic activities hold great promise as active components to propel H_2_O_2_-fueled MNMs [33,34]. The design of H_2_O_2_-fueled MnO_2_-MNMs was first reported in 2014, and since then, diverse Janus-structured Mn-MNMs have been widely reported in various fields, including detection and removal of contaminants, drug delivery, and antibacterial and anticancer [35]. Ge et al. [36] introduced a simple photo-reduction method for fabricating asymmetric bubble-propelled MNMs. The Janus micromotor (JM) is constructed by modifying MnO_2_ nanoflakes on one hemisphere of An-TiO_2_ via photoreduction of KMnO_4_ under aerobic conditions. These MnO_2_ nanoflakes can catalytically decompose H_2_O_2_ to generate O_2_ bubbles, propelling the micromotors at a speed of 48.1 μm s^−1^. Mei and colleagues [37] demonstrated the flexibility of the adopted method by loading 2 types of functional nanoparticles (MnO_2_ and Fe_3_O_4_) onto hydrogel microspheres in a single, direct process (Fig. 1A and B). Subsequently, multifunctional micromotors were efficiently propelled in H_2_O_2_ solution and could also be guided using external magnetic fields. With the increase of the H_2_O_2_ concentration, particles present more extended displacement, which is characterized by more markedly higher mean squared displacement (MSD) (Fig. 1C). If the hydrogel sphere was decorated with MnO_2_ and Fe_3_O_4_ functional nanoparticles in a single process with the mass ratio of 2:1, the obtained multifunctional Janus microspheres present a Brownian motion as shown with the right bottom trajectory (Fig. 1D). With the applied magnetic field, the multifunctional Janus microspheres reveal directional motion. The biotoxicity of MnO_2_ has restricted its applications, leading researchers to explore catalase (CAT) as a biological alternative for powering MNMs [38], particularly in the context of controlled drug release. Chen and colleagues have developed a tadpole-like JN driven by CAT, consisting of a catalytic body and noncatalytic tail (Fig. 1E) [39]. At 2 mM H_2_O_2_ concentration, the nanomotor reaches an average speed of 15.5 μm s^−1^. Additionally, in a simulated tumor microenvironment (TME) constructed with 1.5 mg ml^−1^ collagen, the nanomotor maintains a velocity of 8.0 μm s^−1^. Most of the current artificial nanomotors still have the difficulty in precisely manipulating and modulating the motile behavior on demand. Van Hest and colleagues [40] developed dual-engine Janus supramolecular nanomotors characterized by balanced motion. By coating one side of polymeric bodies with an Au shell, they formed a Janus structure that transformed into wide-neck Janus stomatocytes (Fig. 1F). These nanomotors function through 3 propulsion mechanisms: chemically driven motion via CAT enzymes in the nanocavity decomposing H_2_O_2_ into H_2_O and O_2_, photothermal propulsion with the Au shell activated by near-infrared (NIR) light, and a combined mechanism under simultaneous H_2_O_2_ and NIR light exposure. Notably, when both H_2_O_2_ and NIR light are present, these JNs simultaneously generate 2 competing driving forces, resulting in a “seesaw effect” of balanced motion (Fig. 1G). The seesaw effect is particularly advantageous because it allows for the controlled stalling and resumption of motion, which is achieved by the counteraction between forces—for instance, the NIR-driven force on the Au side can effectively offset the enzymatic propulsion force when applied simultaneously. Crucially, by finely adjusting the incident laser power, researchers can tune the net effect of these 2 forces, granting an unprecedented level of control over the motion behavior (Fig. 1H). This capability is essential for applications requiring precise movement control and positions the seesaw effect as a notable advancement in the field. 

### N_2_H_4_-powered MNMs

The efficiency of H_2_O_2_-driven MNMs is indeed compromised at lower concentrations, which are often present in actual TME [41]. This limitation can lead to insufficient propulsion and hinder the overall performance of MNMs in targeting and treating lesions [42]. A recent study has identified N_2_H_4_ as an attractive alternative, with its remarkably lower fuel concentration threshold of 0.0000001% enabling the powering of MNMs. For example, Gao et al. [43] synthesized SiO_2_-based JMs by coating Ir on one side of SiO_2_ particles using a sputtering deposition method. The Ir catalyzes the decomposition of N_2_H_4_ to produce N_2_, H_2_, and NH_3_ in a gradient that propels the motion of the micromotor (Fig. S3A). At 0.001% fuel concentration, the micromotor exhibits a high average speed of 21 μm s^−1^ (Fig. S3B). The directional movement of JMs can be achieved by depositing a paramagnetic Ni layer, enabling them to respond to magnetic fields. This layer introduces the necessary magnetic anisotropy, allowing the micromotors to experience a mechanical torque, causing them to align and orient themselves in accordance with the magnetic field direction. When an external magnetic field is applied, the Janus motors can be precisely navigated to follow a predetermined trajectory (Fig. S3C and D). Similarly, Wang and colleagues developed a vapor-driven Ir-Au JMs for gas sensing [44], where N_2_H_4_ vapor from the surrounding atmosphere triggers rapid motion (Fig. S3E). Overall, using N_2_H_4_ as a fuel shows promising applications for speeding up motors at low fuel concentrations.

### H_2_O-powered MNMs

Biocompatible fuels are crucial for the application of MNM in medical biology. H_2_O can fulfill this requirement through its reaction with reactive metals. Wang and colleagues [45] synthesized Al–Ga H_2_O-driven JMs, resulting in ejected H_2_ bubbles (Eq. 3) and impressive speeds of 3 mm s^−1^.

$$\mathrm{Al}+H_{2}O\to \mathrm{Al}{\left(\mathrm{OH}\right)}_{3}+H_{2}$$ (3)

The self-degrading nature of H_2_O-driven MNMs eliminates the need for complex recovery procedures. Chen et al. [46] fabricated Mg/ZnO, Mg/Si, and Zn/Fe JMs that produce ions required by living organisms upon reacting with H_2_O (Fig. S3F). For example, Mg reacts with H_2_O to produce H_2_ and propel the motor through bubbles (Eqs. 4) (Fig. S3G).

$$\mathrm{Mg}+H_{2}O\to {\mathrm{Mg}}^{2+}+{2\text{OH}}^{-}+H_{2}$$ (4)

The unique ability of cell derivatives to mimic natural properties has attracted increasing attention. Wu et al. [47] developed cell-mimetic JMs by integrating red blood cell membranes, Au nanoparticle, and alginate into partially embedded Mg particles in parafilm. The exposed area of these Mg particles can react spontaneously with H_2_O in biological media, generating H_2_ bubbles that power the motor.

### Urea-powered MNMs

Urea is a ubiquitous metabolite in mammals, synthesized by the liver and eliminated mainly through urination. Its concentration ranges from 5 to 10 mM in blood but can reach as high as 300 mM in the bladder, rendering it a promising energy source for micromotors to treat bladder-related diseases [48,49]. In contrast to toxic fuels such as H_2_O_2_ and N_2_H_4_, urea offers tremendous biocompatibility, efficiency, and bioavailability advantages, making it popular for driving micromotors. Urease can catalyze the decomposition of urea into NH_3_ and CO_2_, generating a concentration gradient that enables active motion of the MNMs (Eq. 5) [50].

$${\left({\mathrm{NH}}_{2}\right)}_{2}\mathrm{CO}+H_{2}O\to {\mathrm{CO}}_{2}+{2\text{NH}}_{3}$$ (5)

**Fig. 2.**
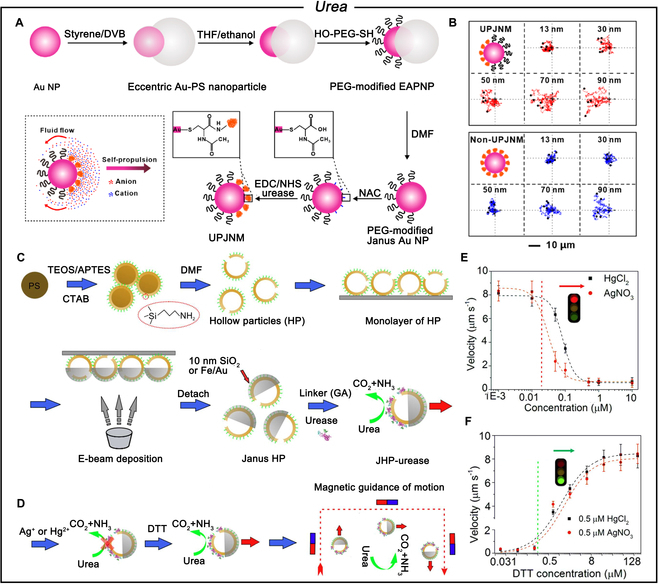
(A) Schematic of the fabrication procedure of UPJNMs. (B) Representative trajectories of the UPJNMs and non-UPJNMs. Reproduced with permission from [52]. Copyright 2023 American Chemical Society. (C) Fabrication process of a JHP-urease micromotors. (D) Motion control on the microcapsule by chemically inhibiting/reactivating the enzymatic activity of urease, and remote magnetic guidance on the movement direction of the micromotors. (E) Velocity of the microcapsule motor at a concentration of 50 mM urea as a function of inhibitor (Ag^+^ or Hg^2+^) concentration. (F) Velocity of the microcapsule motor at 50 mM urea and 0.5 μM inhibitor as a function of dithiothreitol concentration. Reproduced with permission from [53]. Copyright 2016 American Chemical Society.

Sánchez and colleagues [51] have demonstrated the effectiveness of urea-powered mesoporous SiO_2_ nanoparticles in targeting bladder cancer cells. Their findings indicate that these nanomotors can autonomously swim in both simulated and real urine, showcasing substrate-dependent enhanced diffusion. Guan and colleagues [52] developed ultrasmall urease-powered JNs (UPJNMs) composed of an Au nanoparticle core, with one side grafted with polyethylene glycol (PEG) brushes as a biointerface and the other chemically bonded with urease as a propeller (Fig. 2A). Their size can be precisely tuned from approximately 30 to 100 nm. As the size of the UPJNMs increases, the tracking paths exhibit an increase in positive chemotactic shift. However, for the control group without urea concentration gradient, the UPJNMs only show an irregular enhanced diffusion (Fig. 2B). Ma et al. [53] developed Janus hollow mSiO_2_ microparticles with immobilized urease on one side to drive the particles via self-diffusiophoresis (Fig. 2C). The speed of the urease-conjugated Janus hollow particle (JHP-urease) could be controlled through the addition of solid inhibitors like Ag^+^, Hg^2+^, or thiol-protective reagent dithiothreitol to manipulate the enzymatic activity of urease. The multilayer assembly of the enzyme on Janus magnetic particles allows the urease to actuate the micromotor, making it responsive to the magnetic field and controlling its direction of motion (Fig. 2D). Moreover, when the concentration of inhibitor reaches 0.5 μM, the JHP-urease can be slowed down and stopped (Fig. 2E). In contrast, the speed increases rapidly with the addition of dithiothreitol (Fig. 2F). The maximum speed achieved by these micromotors is only about 10 μm s^−1^ at 25 mM urea concentration. Further speed improvements will be beneficial for overcoming the complex, dense environment of the human body. Guan and colleagues [54] demonstrated a considerable enhancement in the propulsion speed of Janus Au/magnetic micromotors through the multilayered self-assembly of enzymes, achieving a velocity of 21.5 μm s^−1^ at physiological urea concentrations (10 mM). Previous reports have employed multiple cell models to evaluate the application of urease-driven nanomotors in cancer therapy, demonstrating the feasibility of this propulsion mechanism. It is important to consider that in vivo, there is a potential for nonspecific adhesion of the nanomotors to other cell surfaces. This nonspecific binding could lead to increased phagocytosis, thus reducing both the utilization efficiency and safety of the nanomotors. In future studies, it would be beneficial to focus on developing improved design strategies to minimize the risk of nonspecific adhesion, thereby potentially enhancing the specificity and effectiveness of these nanomotors in clinical applications.

Prior micromotors incorporating inorganic compounds or metals exhibited limited biocompatibility. Natural cells emerge as promising candidates as carriers of urease, benefiting from their exceptional biocompatibility and facile metabolism [55]. Wang and colleagues [56] synthesized Janus platelet micromotors (JPL-motors) by partially loading urease onto the platelet (PL) surfaces. PLs were attached to a modified poly(l-lysine) 12-well plate, partially blocking their surfaces and allowing the remaining unblocked surfaces to be modified with urease via a biotin–streptavidin–biotin binding complex (Fig. S4A). In vitro drug delivery was investigated with breast cancer cell MDA-MB-231. The binding of JPL-motors to MDA-MB-231 cells was observed and verified through fluorescent staining (Fig. S4B). The transformation of PL into JPL nanomotors does not negatively impact the rate of drug release at similar pH levels (Fig. S4C). Moreover, Fig. S4D illustrates that after different coculture periods with cells, the active motion of the JPL nanomotors accelerates the specific adhesion to cancer cells compared to PL. Previous literature also suggests that the motorized movement increases the collision probability between the nanoparticles and the cell membrane, leading to enhanced specific adhesion of nanomotors to cancer cells compared to passive diffusion nanoparticles [24].

### Glucose-powered MNMs

Glucose is a vital energy source for human cells, and it can be catalyzed by glucose oxidase (GOx) to produce glucuronic acid and H_2_O_2_ [57,58], which can be decomposed by CAT to power Janus MNMs [59,60]. To increase the efficiency of catalytic reactions, MNMs with glucose as fuel require 2 different catalysts for the catalytic decomposition of glucose and the intermediate product of H_2_O_2_ [61,62]. The decomposition of H_2_O_2_ has been discussed in detail in the previous chapter. One approach to improve the efficiency of glucose-powered MNMs is to incorporate different biological enzymes within their unique structure [63]. For instance, Städler and colleagues [64] assembled submicrometer-sized JPs with one hemisphere decorated with GOx and CAT, using glucose as fuel, which exhibited enhanced diffusion behavior dependent on glucose concentration. Another strategy involves integrating enzymes and metallic catalysts into a single nanostructure, thereby producing chemical cascades. Examples include the GOx and Pt nanoparticles that drive the movement of Janus swimmers by catalyzing the decomposition of glucose to H_2_O_2_ and further breaking down H_2_O_2_ to harmless H_2_O and O_2_ (Fig. S4E) [9]. The diffusion properties of the Janus swimmers can be synergistically enhanced when the peptide-fueled trypsin engine was further combined with the engine mentioned above on the same carrier (Fig. S4F). Further deposition of multiple layers of magnetic manganese ferrite nanoparticles on the surface of the swimmer (Fig. S4G to I) allows the swimmer to move in a magnetic gradient when an external magnetic field was applied, thus producing a directional motion of the dual-fuel Janus swimmer (Fig. S4J). In addition, Ji et al*.* [65] reported GOx-driven Janus Au nanoswimmers functionalized with polymer brushes. These nanoswimmers were synthesized by grafting polymer brushes onto one side of Au nanoparticles and functionalizing the opposite side with GOx (Fig. S4K). Figure S4L illustrates that the average speed of PNIPAM@JAu@GOx nanoswimmers increased from 1.2 μm s^−1^ in a 5 mM glucose solution to 9.1 μm s^−1^ in an 80 mM glucose solution. In comparison, JAu@GOx nanoswimmers (without polymer brushes) displayed a similar trend but exhibited lower speeds at the same glucose concentrations. These findings suggest that the polymer brushes substantially enhance the translational diffusion of Janus Au nanoswimmers (Fig. S4M). In the study by Kwon et al*.* [58], Au/Pt-based Janus nanostructures, which exhibit an “egg-in-nest” morphology (Au/Pt-ENs), are introduced to demonstrate enhanced motion due to a dual enzyme-relay-like catalytic cascade occurring in physiological biomedia. The unique anisotropic nest-like morphology, featuring intimately interfaced Au and Pt catalytic sites, enables these nanomotors to effectively oxidize glucose and generate O_2_ gas. The velocity of the Au/Pt-EN nanomotors increases with glucose concentration, as higher glucose levels enhance the chemical gradients that strengthen their self-propulsion capabilities. This increased diffusiophoresis in cell culture media enhances nanomotor–membrane interaction events, thereby facilitating cell internalization.

## External Field-Powered Janus MNMs

The chemical propulsion mechanism in MNMs operates by catalytically decomposing additional chemical fuels [66]. Despite the effectiveness of this method, the insufficient fuel concentrations in practical environments poses a substantial limitation for biomedical applications. This limitation can lead to insufficient propulsion and hinder the overall performance of MNMs in targeting lesions. To address these issues, an alternative approach is the implementation of fuel-free propulsion mechanisms. These mechanisms utilize external stimuli, such as light, magnetic fields, ultrasound, and thermal energy, to propel the motors, thereby expanding their potential applications.

### Light-driven MNMs

Light has emerged as a favored external stimulus for MNM propulsion, obviating the requirement for toxic chemical fuels. The development of light-powered MNMs, which utilize photocatalytic or photoactive materials, enables the efficient conversion of light energy into mechanical motion [67]. A key factor in the movement of light-powered JMs is the creation of an asymmetrical gradient field generated by light. By adjusting the intensity, frequency, and polarization of the light, we can influence the speed and direction of these micromotors in a liquid environment, opening promising applications across various fields. This section highlights NIR-, visible-, and ultraviolet (UV)-driven MNMs, detailing their distinct characteristics [68,69].

### NIR light-propelled MNMs

**Fig. 3.**
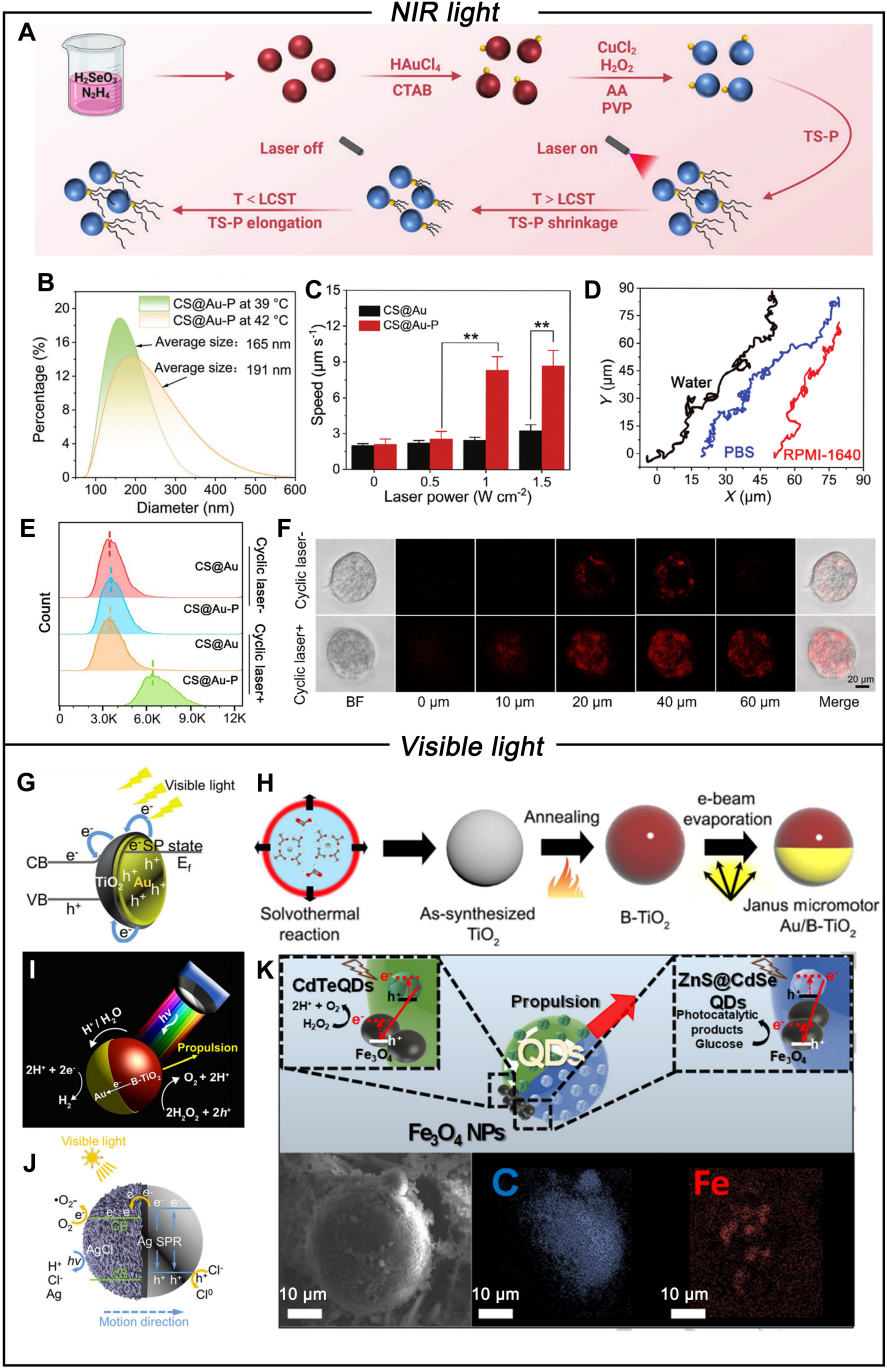
(A) Schematic illustration of the preparation and motion mechanism of CS@Au-P nanomotors. (B) Hydrodynamic diameters of CS@Au-P nanomotors at 39 and 42 °C. (C) Speeds of Cu_2−*x*_Se@Au-P nanomotors and CS@Au nanoparticles under different NIR power densities. (D) Tracking trajectories of CS@Au-P nanomotors under different NIR power densities. (E) Representative histogram of KYSE30 cells incubated with CS@Au-P and CS@Au without or with cyclic laser irradiation by flow cytometry. (F) Colocalization of bright-field and Z-stack fluorescence images using confocal microscopy of CS@Au-P nanomotor penetration into 3D multicellular tumor spheroid (MTS) in the presence or absence of cyclic laser irradiation, respectively. Reproduced with permission from [77]. Copyright 2024 Wiley-VCH GmbH. (G) Schematic cartoon for the surface plasmon resonance effect of Au/TiO_2_ under visible light. Reproduced with permission from [79]. Copyright 2018 Wiley-VCH Verlag GmbH & Co. KGaA, Weinheim. (H) Synthesis of Au/B-TiO_2_ JMs. (I) Schematic of the propulsion mechanism of Au/B-TiO_2_ JMs. Reproduced with permission from [80]. Copyright 2017 American Chemical Society. (J) Schematic image for visible light absorption process of Janus PS/Ag/AgCl micromotors based on the surface plasmon resonance effect and model of the visible light actuated motion. Reproduced with permission from [86]. Copyright 2018 American Chemical Society. (K) Schematic of the propulsion mechanism of quantum dot–Fe_3_O_4_ light-driven micromotors in H_2_O_2_ and glucose media. Reproduced with permission from [87]. Copyright 2019 The Authors. Published by Wiley-VCH Verlag GmbH & Co. KGaA, Weinheim.

NIR light represents a compelling stimulus for propelling motors, owing to its capacity to enable wireless and remote energy control, penetrate deep tissues, and achieve reversible ON/OFF light switching [70,71]. NIR-driven MNMs enable noninvasive manipulation with high-precision spatiotemporal control. Among various photothermal materials, Au nanoshells are particularly notable for their ability to generate a photothermal effect via surface plasmon resonance under NIR irradiation. This capability allows for the precise ablation of cancer cells through photothermal therapy (PTT) and thermophoresis [72,73]. The presence of an asymmetric gradient field generated by the photothermal process induces a microflow that propels the motor toward equilibrium [68,74]. To fabricate nanomotors with a Janus structure composed of Au, a commonly employed method involves distributing nanoparticles into a monolayer film on a substrate, followed by deposition of Au onto half the nanoparticle surface [14]. Typically, van Hest and colleagues [75] prepared biodegradable poly (ethylene glycol)-b-poly (D, L-lactide) (PEG-PDLLA) block copolymer nanospheres, followed by the modification of hemispherical Au nanoshell through sputter coating. This Janus morphology allows such hybrid polymersomes to undergo photothermal motility in response to thermal gradients generated by plasmonic absorbance of NIR light irradiation, with velocities ranging up to 6.2 μm s^−1^. In addition, particle motion switched from Brownian to directional concurrently upon laser irradiation. Deng et al. [76] also synthesized magnetron-sputtered MNMs with swarming behavior and collective migration by the asymmetric coating Pt on one side of a TiO_2_ sphere layer. Upon NIR irradiation, a temperature gradient is induced, triggering convection flows that drive the MNMs’ collective swarming and navigation. The above structure efficiently converts NIR light into heat, generating a thermal gradient across the nanoparticle surface, which induces thermophoretic forces that drive the motion of the nanomotor. Differently, Cheng and colleagues [77] initially proposed the concept of nanomotors driven by the expansion and contraction of thermosensitive polymers. The JNs (CS@Au-P) were engineered through controlled asymmetric growth of Au nanoparticles on the Cu_2−*x*_Se surface, followed by the attachment of thermosensitive polymers via Au-S bonds (Fig. 3A). Due to the photothermal effect of Cu_2−*x*_Se, the system’s temperature can be controlled by an NIR laser, inducing alternating expansion and contraction of the thermosensitive polymer structure (Fig. 3B). This dynamic transformation acts as the driving force of the nanomotor, facilitating a swimming-like motion. The motion of the CS@Au-P nanomotor can be controlled by the power density of the NIR laser (Fig. 3C and D). NIR light can control the initiation and cessation of MNM movement and activate PTT to selectively ablate tumors. The enhanced therapeutic effect of CS@Au-P nanoparticles is achieved through increased cellular uptake (Fig. 3E) and tumor penetration (Fig. 3F). For MNMs composed of noble metals such as Au and Pt, they exhibit high resistance to oxidation due to their chemically stable surface properties. This stability reduces the release of ions into biological environments, demonstrating relatively good biocompatibility.

### Visible light-driven MNMs

Visible light is a promising external stimulus for the propulsion of MNMs due to its good biocompatibility, broad-spectrum coverage, and deep tissue penetration depth [78]. Wang et al. [79] fabricated a Janus TiO_2_/Au nanomotor by combining TiO_2_ with Au using plasma etching and PVD methods. When illuminated with a white halogen cold light source, the Brownian motion of the nanomotor can be increased by about 0.8 times, and the enhanced movement results from the self-electrophoresis between the Au and TiO_2_ layers (Fig. 3G). Similarly, Pane and colleagues [80] developed multi-wavelength light-responsive JMs by coating a thin Au layer on a black TiO_2_ hemisphere (Fig. 3H). When the uncoated Au hemisphere was exposed to light, this JM moved through a self-diffusion electrophoretic mechanism (Fig. 3I). Bismuth iodide (BiOI) microspheres can also be activated by visible light. Dong et al. fabricated BiOI JMs by coating a metal film on the hemispheres of BiOI microspheres [81], which are based on a self-electrophoretic propulsion mechanism powered by photocatalytic reactions using visible light and H_2_O as energy sources [82]. The micromotor’s motion changes from Brownian to directional movement when the light intensity increases to a certain level. The Ag/AgCl composites also have strong absorption in both visible and NIR regions because of the coupling to the surface plasmon resonance of Ag [83]. Makarov et al. demonstrated blue light-driven Ag/AgCl-based spherical JMs [84] that couple plasmonic light absorption with the photochemical decomposition of AgCl [85]. These micromotors reveal high motility in pure H_2_O, i.e., MSDs reaching 800 μm^2^ within 8 s, which is 100 times higher compared to previous visible light-driven BiOI JMs [82]. Besides, preferable velocity was realized by a self-diffusion electrophoretic mechanism for the Ag/AgCl JMs [86]. Figure 3J displays the photocatalysis process and motion direction of Ag/AgCl JMs. Under the irradiation of visible light, AgCl at the location of the cap will be reduced and release Cl^−^ and H^+^ ions. The different diffusion speeds of H^+^ and Cl^−^ ions induce an asymmetric internal electric field around JMs, propelling the JMs to move toward the smooth polystyrene surface, and its speed can be adjusted by varying the light intensity. Pacheco et al. [87] assembled JMs using polycaprolactone encapsulation of CdTe or CdSe@ZnS quantum dots as photoactive materials and an asymmetric Fe_3_O_4_ patch for propulsion. The structure and propulsion mechanism of micromotors in H_2_O_2_ and glucose media are shown in Fig. 3K. The JMs absorb visible light, which excites electrons from the valence band to the conduction band in quantum dots, creating holes in the valence band. These holes then interact with H_2_O_2_ or glucose, initiating complex radical chain reactions. These reactions produce various products such as H^+^ and O_2_ in H_2_O_2_ media, or arabinose, erythrose, and formic acid in glucose media [88]. The accumulation of these photodegradation products around the JMs surface enhances propulsion. These visible light-driven MNMs show promising prospects for various applications.

### UV light-propelled MNMs

The application of UV light as an external stimulus notably enhances the propulsive force of MNMs, making it a highly effective mechanism for their propulsion [89]. Various materials, including WO_3_@C [90], TiO_2_ [91], and metal/TiO_2_ composites [92], have been employed in the fabrication of UV-driven MNMs. Zhang et al*.* [90] developed UV light-driven JMs by depositing an Au layer onto colloidal WO_3_@C nanoparticle composite hemispheres (Fig. S5A and D). When the surface of a WO_3_@C is irradiated with UV light, a negative electron (e^−^) and H^+^ pair created by WO_3_ can react with H_2_O or O_2_ to induce complex radical chain reactions and produce various products such as H_2_O_2_, H^+^, OH^−^, and O^2−^, which allows the JMs to move at 16 μm s^−1^ by a self-diffusion mechanism (Fig. S5E). Dong and colleagues developed TiO_2_-based JMs by depositing Au and Ni layers on one-half of the particles (Fig. S5F). The presence of a paramagnetic Ni layer provides the necessary magnetic properties that enable the nanomotor to be guided along a predetermined trajectory when subjected to an external magnetic field. UV light exposure drives these micromotors via a redox reaction that generates a proton flux and electric field. The deposited paramagnetic Ni layer ensures that the motor’s trajectory can be easily controlled and adjusted in real time using magnetic fields, thus achieving precise directional movement (Fig. S5G). Light intensity or chemical fuel concentration could regulate the micromotor’s activation, acceleration, deceleration, and stopping [91]. Figure S5H presents Janus hollow mesoporous TiO_2_/Au (JHP–TiO_2_–Au) micromotors with enhanced swimming speeds under low-intensity UV light by self-electrophoresis [93]. JHP–TiO_2_–Au micromotors presented enhanced swimming speeds under low-intensity UV light due to their porous structure, which allowed photoelectrons to migrate to the surface more efficiently. Wang et al*.* [94] advanced the field of nanomotors by fabricating TiO_2_–Fe JMs, integrating the cost-effective metal Fe with the conventional photocatalyst TiO_2_. Fe was implemented as an electron-absorbing layer to enable self-electrophoretic motion and direction-controlled response to an external magnetic field. Maric and colleagues investigated the influence of various metals on the velocity of nanomotors, including Pt/TiO_2_, Cu/TiO_2_, Fe/TiO_2_, Ag/TiO_2_, and Au/TiO_2_ JMs, under UV radiation in pure H_2_O (Fig. S5I) [92]. Their study demonstrated that the interplay of chemical potential and catalytic activity pronouncedly affect the speed of these micromotors. The Pt/TiO_2_ JMs exhibited the highest speed under identical external conditions. In addition, the velocity of the Fe/TiO_2_ nanomotors was greater than that of Au/TiO_2_ (Fig. S5J). The enhanced velocities of Pt/TiO_2_ and Fe/TiO_2_ are attributed to the synergistic effects of its chemical potential and catalytic activity.

### Magnetically propelled MNMs

The propulsion of nanoparticles with magnetic fields has been widely acknowledged, particularly when exposed to a gradient or rotating field [95,96]. Given their advantageous characteristics of high penetration, noninvasiveness, and precise navigation capabilities, magnetic fields have been identified as attractive methods for driving MNMs in biomedical applications such as targeted drug delivery and cancer therapy [97,98]. Modification of MNMs with ferromagnetic components enables their propulsion under the influence of an external magnetic field without the need for additional chemical agents. Previous studies have shown that a spiral-type tail combined with a macroscopic magnetic object and a rotational magnetic field can result in the self-propulsion of the device in the liquid environment [98]. Moreover, the direction and speed of the motor can be remotely and precisely controlled by an external magnetic field [99]. Co and Ni are widely used elements for magnetic-driven nanomotors, and they are typically modified on the motor through PVD [100,101]. Schmidt and colleagues [102] developed SiO_2_-based Janus motors combining catalytic and magnetic caps structures and achieved deterministic motion. This magnetic configuration affords precise control over JP motion, demonstrating exceptional performance in manipulation, loading, transport, and delivery. Chen et al. [103] synthesized JMs by coating SiO_2_ microspheres with CoFe_2_O_4_–BaTiO_3_ bilayer composite hemispheres. They fabricated self-assembled monolayer SiO_2_ microspheres on a silicon wafer, followed by coating with magneto restrictive CoFe_2_O_4_ layer and piezoelectric BaTiO_3_ layer using magnetron sputtering. The inner magnetic CoFe_2_O_4_ layer enabled the micromachines to be maneuvered using low-magnitude rotating magnetic fields, while the magnetoelectric bilayer composite allowed for remote generation of electric charges upon application of a time-varying magnetic field. This integrated micromachine can be used for on-demand functions driven by magnetic energy using a simplified operating system. Li and colleagues aimed to develop a more straightforward configuration to power magnetic JMs inspired by microbial and cellular motility [104]. The new microdimer surface walker consists of 2 magnetically connected Ni/SiO_2_ (or polystyrene) Janus microspheres produced by coating a thin layer of Ni (Fig. S5K and L). The corresponding scanning electron microscopy (SEM) images of JPs are shown in Fig. S5M and N. These miniature motors can achieve a velocity of 18.6 μm s^−1^ in an oscillating magnetic field at 25 Hz, performing fast and accurate magnetic steering in complex environments. In general, magnetic fields have unique advantages for the speed and direction control of MNMs, allowing them to be used in conjunction with other dynamic fields. However, these types of nanomotors often contain more reactive elements like Co or Ni. In biological environments, such materials can release ions like Ni^2+^, which are known to pose risks of cytotoxicity and may trigger immune responses [105], thus negatively impacting their biocompatibility.

### Ultrasound-powered MNMs

**Fig. 4.**
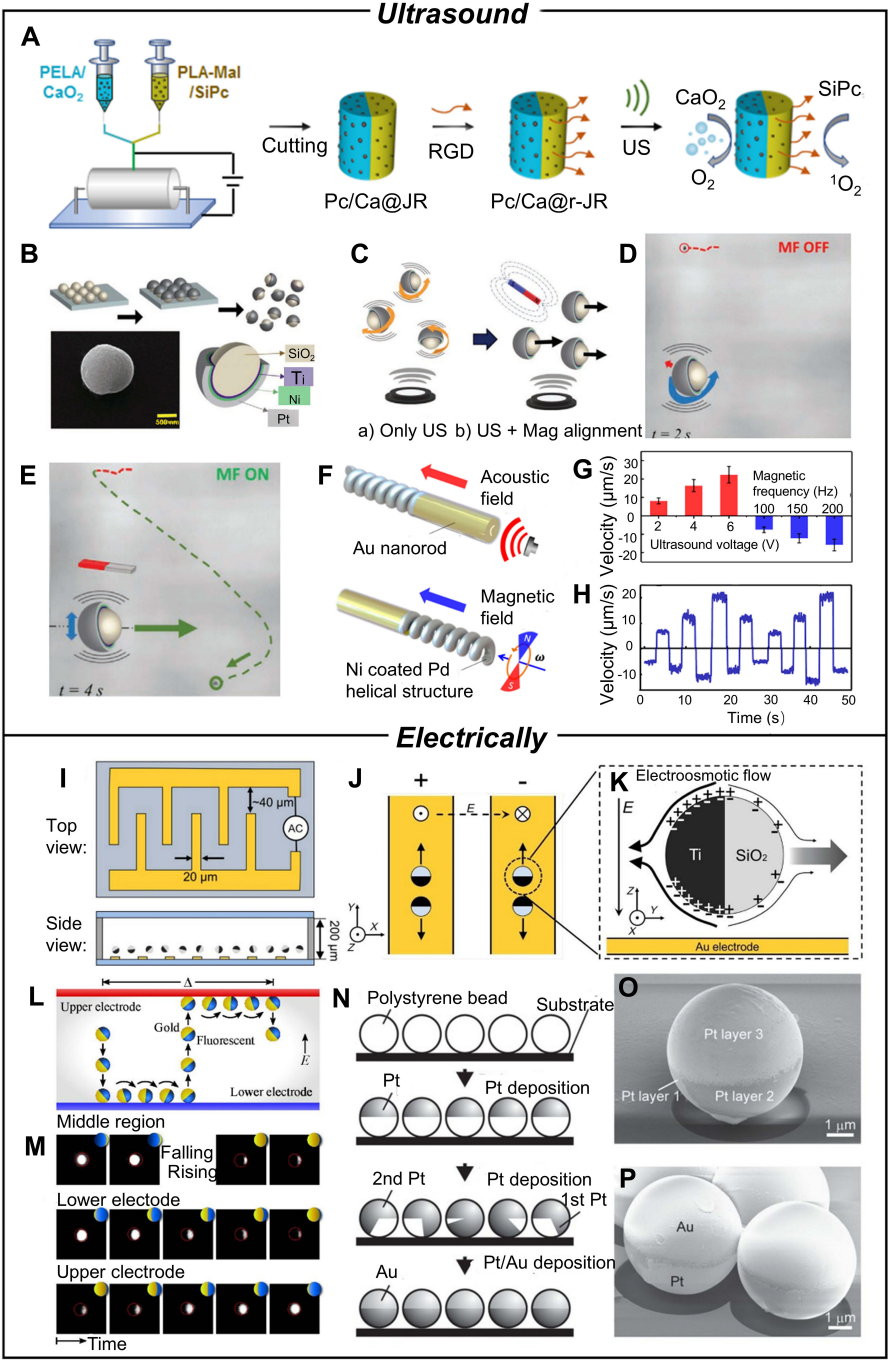
(A) Aligned fibers with the Janus structure are prepared by side-by-side electrospinning, and cryocutting was used to fabricate Janus rods with designed aspect ratios, followed by Arg–Gly–Asp grafting to obtain Pc/Ca@r-JRs. Reproduced with permission from [108]. Copyright 2021 American Chemical Society. (B) Scheme of fabrication of Janus microstructures by thin-film deposition cross-section of the Janus structure composition. (C) Scheme of JMs behavior without and with fixed magnetic field orientation. (D and E) Spinning without a magnetic field and propulsion with a static magnetic field. Reproduced from [109] with permission from Wiley-VCH GmbH, Copyright 2020. (F) Scheme of the design of the magneto-acoustic hybrid nanomotor and its dual propulsion modes under the acoustic and magnetic fields. (G) Quantitative velocity of the hybrid motor using magnetic frequencies of 100, 150, and 200 Hz and ultrasound voltage amplitudes of 2, 4, and 6 V. (H) Digital control of the hybrid motor speed by changing the actuation mode and the corresponding ultrasound voltage and rotational magnetic frequency alternately every 3 s using the following sequence. Reproduced with permission from [110]. Copyright 2015 American Chemical Society. (I) Schematics of the top and side view of a chip. (J) Top view of the direction and orientation of JPs moving on electrodes. (K) Schematic of a SiO_2_/Ti particle undergoing induced charge electrophoresis. Reproduced with permission from [114]. Copyright 2019 American Chemical Society. (L) Schematic illustration of the propulsion mechanism showing one oscillation cycle. (M) Fluorescent microscopy images highlight the nonmetallic hemispheres of fluorescent JPs. Reproduced with permission from [115]. Copyright 2016 American Chemical Society. (N) Fabrication process of micromotors. (O) SEM images of PS/Pt particles. (P) SEM images of PS/Pt/Au particles. Reproduced with permission from [116]. Copyright 2015 Wiley-VCH Verlag GmbH & Co. KGaA, Weinheim.

This section reviews recent advancements in utilizing ultrasound technology to control and propel the motion of Janus-structured MNMs, a versatile class of motors characterized by customizable motion behavior, extended lifetimes, noninvasiveness, contact-free operation, and deep tissue penetration [106]. Ultrasound waves generate pressure gradients through the formation of nodes and antinodes, allowing modulation of motor movement via adjustments in ultrasound frequency, direction, or on/off cycles of the ultrasonic source. Furthermore, MNMs can be captured and transported through transverse motion by intermittently toggling acoustic waves. Employing ultrasound for propulsion presents a promising alternative, enabling noninvasive and biocompatible interactions between nanomotors and biological systems [107]. For example, Li and colleagues [108] synthesized Janus rod-shaped micromotors for sonodynamic therapy of thrombosis, using silicon phthalocyanine and CaO_2_ nanoparticles loaded into separate sides of Janus rods and Arg–Gly–Asp peptides grafted onto the surface (Fig. 4A). Ultrasonication of O_2_ bubbles generated by decomposition of CaO_2_ nanoparticles produces cavitation effect, driving the thrombus penetration. Another study showed that density asymmetric JMs can be translationally moved by using an external magnetic field [109]. The JMs were fabricated by depositing a Ti layer onto SiO_2_ beads, followed by a Ni layer for magnetic orientation, and a high-density Pt layer to induce a substantial density asymmetry (Fig. 4B and C). Under ultrasound radiation alone, the JMs exhibited a low speed of 5 mm s^−1^ (Fig. 4D). However, with the alignment of the Ni segment with magnetic field lines, the JMs present higher propulsion with a speed of 35 mm s^−1^ in a straight-line direction (Fig. 4E). Wang and colleagues [110] developed a magneto-acoustic hybrid fuel-free nanomotor based on a bisegment configuration, with a magnetic nanospring (Ni-coated Pd) on one end serving for magnetic propulsion and a concave (Ni-coated Au) nanorod on the other for acoustic propulsion (Fig. 4F). The obtained Janus motor has reversible swarming states and collective actions that cannot be achieved using a single propulsion mode. Under an applied voltage of 6 V, the motor attains a speed of 22.3 μm s^−1^, while the hybrid motor, which operates in magnetic mode, yields a speed of 15.9 μm s^−1^ at a rotational frequency of 200 Hz (Fig. 4G and H). The nanomotor displays reversible swarming states and collective actions, indicating its suitability for implementation in high ionic strength biological environments.

### Electrically propelled MNMs

Similar to light and magnetic field, electric fields are capable of driving various MNMs. Conducting materials are generally required for electric field-driven MNMs to respond effectively to the field [111]. Due to coulomb interactions, charged particles move toward opposite-charged electrodes, which permit the use of alternating current (AC) electric fields to regulate particle motion. Researchers have pioneered several types of electric field-driven Janus MNMs, which have demonstrated the ability to control movement along specific directions using different frequencies or polarizations of electric fields. These findings hold great potential for applications in sensing, sorting, transportation, and in vitro cell manipulation or drug delivery [112]. Yan et al. [113] developed a Si sphere-based nanomotor with a metal coating and incidental SiO_2_ protective layer. This motor leveraged the distinct polarizations of the metal and Si hemispheres, thereby enabling the generation of ionic flows through modulation of the localized electric field strength for propulsion purposes. Moreover, adjustments in the frequency of the electric field provided effective control over both the direction and velocity of the motor’s motion. Inspired by the concept of micromotors walking on a one-dimensional (1D) track, Wang and colleagues [114] devised a strategy for confining, advancing, and collecting metal–dielectric JMs using an interdigitated microelectrode design (Fig. 4I). The motor is kept in the center of the electrode by the horizontal component of the AC electric field and propelled in a 1D direction toward the end of the electrode through induced charge electrophoresis by the vertical component of the field (Fig. 4J and K). This noncontact manipulation strategy demonstrates the potential for basic research in active substances and various applications such as sensing, sorting, and transportation. Dou et al. [115] explored the dynamics of metal–dielectric JPs in motion using contact charge electrophoresis between parallel electrodes. They proposed a mechanism based on particle rotation caused by charge transfer at the electrode surface, which results in particle translation. The JPs exhibited rapid oscillations between the electrodes, reaching a velocity of 600 μm s^−1^. Furthermore, interactions between multiple particles resulted in repulsive, attractive, or synergistic motion depending on the position and phase of individual oscillators (Fig. 4L to N). In a separate study by Peyrade and colleagues [116], an Au/Pt bilayer JM was created by depositing Pt layers on polystyrene spheres for distribution throughout the sphere and Au layers on the hemispheres to form the Janus structure (Fig. 4O and P). The micromotor’s 2D movement was achieved using AC electro-osmosis and positive dielectric electrophoresis with an AC electric field. These conditions caused the PS/Pt/Au micromotor to be dragged by AC electro-osmosis and positive dielectric electrophoresis to the middle of the indium tin oxide wire electrode while moving along the top and middle surface of the activation electrode. The fluctuation of the voltage applied to the electrodes led to an adjustment of individual or collective self-propelled micromotor orientation.

## Application of Janus MNMs

The application of Janus MNMs has garnered attention in the field of biomedical research due to their unique anisotropic structure, diverse composition, and remarkable performance in a wide range of applications [117–120]. In this section, we delve into the specific application of Janus MNMs in biomedical research, with a particular emphasis on their role in cancer therapy, bioimaging, and biosensing.

### Cancer therapy

**Fig. 5.**
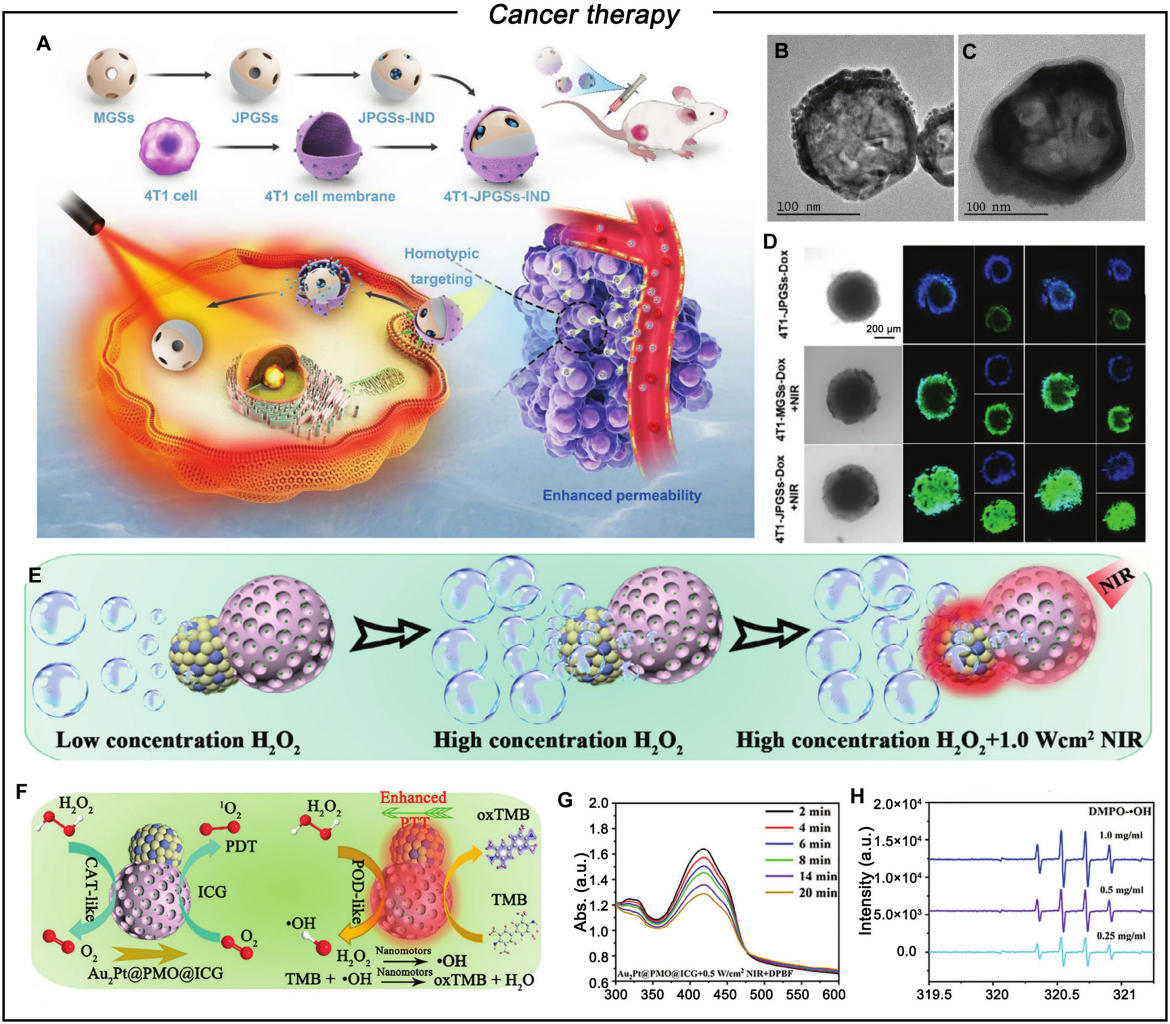
(A) Fabrication process of biomimetic 4T1-JPGSs-IND and its effect on enhancing PTT and alleviating PTT-induced inflammation. Transmission electron microscopy (TEM) images of (B) JPGSs and (C) 4T1-JPGSs-IND. (D) Enhanced penetration of 4T1-JPGSs-IND under NIR illumination in MTSs. Reproduced from [128] with permission from Wiley-VCH GmbH, Copyright 2023. (E) Schematic diagram of APIJNS drive. (F) Schematic diagram of the catalytic process of APIJNS. (G) UV–visible absorbance at 808 nm of the ICG solution supernatants and APIJNS supernatant. (H) Electron spin resonance spectra of •OH captured by 5,5-dimethyl-1-pyrroline-n-oxide (DMPO) for different concentrations of APIJNS solutions in an acidic environment. Reproduced from [138] with permission from Wiley-VCH GmbH, Copyright 2024.

This section highlights the use of MNMs for eradicating cancer cells. Drug delivery has been recognized as a successful therapeutic approach. Micromotors containing specific materials and functionalities can directly eliminate these tumor cells upon contact [121,122]. Among these techniques, the Janus MNMs with the PTT effect have gained considerable popularity, employing light energy to generate heat energy, thereby effectively eliminating cancer cells with minimal adverse effects and remarkable tumor targeting [123–127]. Tu and colleagues [128] prepared NIR light-driven JNs loaded with indomethacin (IND), which were subsequently decorated with 4T1 cancer cell membrane (CCM) vesicles. The preparation process and the morphology of Janus Pt–Au nanospheres (JPGS) and 4T1-Janus Pt–Au nanospheres-IND (4T1-JPGS-IND) are illustrated in Fig. 5A to C. Under laser irradiation, these nanomotors generate heat via the Au nanoshell, which enhances their diffusion through the self-heating effect. The semi-deposited Pt in the Janus structure facilitates unidirectional movement of the nanomotors. This thermomechanical penetration effect, along with the modification by 4T1 CCM vesicles, enhances the specific interaction between the nanomotors and tumor tissue, allowing the 4T1-JPGS-IND nanomotors to penetrate deeper into the tumor tissue (Fig. 5D), thereby improving the PTT efficacy through ablation at deeper tumor layers. Furthermore, the incorporation of IND helps to mitigate the inflammatory responses usually caused by PTT, enhancing therapeutic effectiveness. Photodynamic therapy (PDT) has emerged as a highly promising cancer treatment, attracting attention in recent years [129,130]. PDT employs light-activated photosensitizers to transfer energy to oxygen molecules (^3^O_2_), generating cytotoxic singlet oxygen (^1^O_2_) that effectively eradicates cells or bacteria. However, limited ^3^O_2_ availability around the photosensitizer and the short diffusion range of light-activated ^1^O_2_ hinder effective PDT efficiency [131,132]. Ma and colleagues [133] developed a Janus enzymatic micromotor to address these concerns using hollow mSiO_2_ microspheres as a carrier. The inherent asymmetry of mSiO_2_ spheres enables ion diffusion electrophoresis through urea enzymatic digestion, driving active micromotor propulsion. By integrating autonomous self-propulsion, this system overcomes the limitations of PDT by improving the accessibility of the photosensitizer to ^3^O_2_ and expanding the ^1^O_2_ diffusion range, ultimately improving the efficacy of PDT treatments. However, the successful implementation of PTT and PDT as promising therapeutic approaches is hindered by several challenges, including limited accumulation and penetration of photosensitizers within tumors and dose-dependent side effects that can negatively impact patient outcomes [134,135]. Li and colleagues [136] addressed these limitations by developing persistent luminescence-activated nanoparticles that effectively combined PTT and PDT using persistent luminescence. The complex TME is characterized by severe hypoxia, H_2_O_2_ accumulation, and dense physical properties, which limit the efficacy of nanomedicines in tumor treatment. Synthetic MNMs have demonstrated versatility in modulating abnormal TME and overcoming the limited penetration of solid tumors. Zheng and colleagues [137] constructed chemical-NIR dual-propelled IR820@CuS/Pt JNs by depositing Pt onto the surface of CuS and encapsulating IR820. The deposited Pt effectively catalyzes the conversion of endogenous tumor H_2_O_2_ into O_2_, which extremely relieved the tumor hypoxia state and allowed the chemical propulsion of nanomotors. Such autonomous motion remarkable improved the tumoral accumulation of nanomotors and enhanced cellular uptake and deep tumor penetration. Additionally, the ample oxygen supply promotes in situ reactive oxygen species (ROS) generation in the tumor, thereby enhancing the efficacy of PTT/PDT. H₂O₂ is a ROS that can have cytotoxic effects on cells in high concentrations. The current study mitigates this concern by employing NIR-driven motion to regulate the nanomotor’s activity and distribution in vivo experiment. Chen and colleagues [138] synthesized dual-source driven parachute-shaped Au_2_Pt@PMO@ICG JNs (APIJNS) through an interfacial energy-mediated anisotropic growth strategy for PTT-mediated triple synergistic cancer therapy combining chemodynamic therapy (CDT) and PDT (Fig. 5E). As shown in Fig. 5F, under 808-nm NIR laser irradiation, a thermal gradient is generated between the Au_2_Pt and periodic mesoporous organosilica (PMO) structures due to the excellent and stable photothermal properties of the Au_2_Pt enzyme, achieving self-thermophoretic propulsion. Additionally, the Au_2_Pt enzyme exhibits high CAT activity, efficiently catalyzing H_2_O_2_ to produce O_2_ and enabling autonomous propulsion. The O_2_ produced through catalytic decomposition is converted into ^1^O_2_ and utilized by the photosensitizer indocyanine green (ICG) to enhance PDT efficiency (Fig. 5G). Furthermore, the Au_2_Pt enzyme possesses high peroxidase (POD)-like catalytic activity, which catalyzes the decomposition of H_2_O_2_ to generate highly toxic ·OH (Fig. 5H), thereby enhancing H_2_O_2_-induced CDT efficiency within the TME.

### Bioimaging

**Fig. 6.**
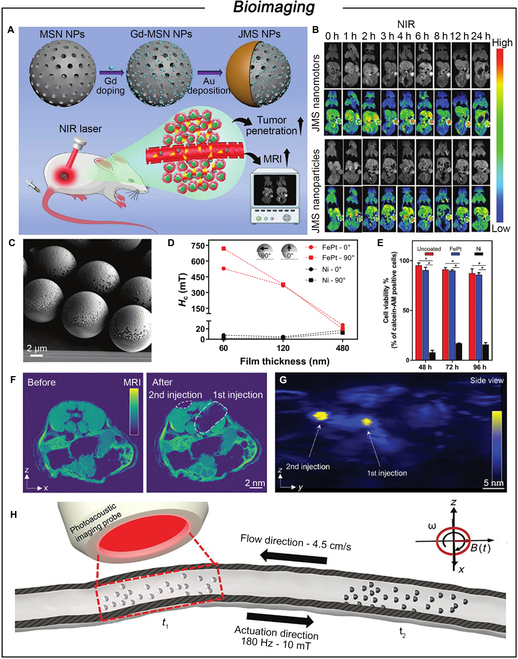
(A) Schematic illustration of the preparation process of JNs and the NIR-powered Janus mSiO_2_ nanoparticles for tumor deep penetration and enhanced MRI. (B) T1-weighted MRI of mice model. Reproduced from [145] with permission from Wiley-VCH GmbH, Copyright 2021. (C) SEM image of the 60-nm-thick FePt-coated microrollers. (D) Vibrating sample magnetometer (VSM) characterization results of FePt-L10 and Ni coating at different thicknesses (60, 120, and 480 nm). (E) The % viability of the cell–particle complexes that engulfed the particles was quantified based on their calcein-AM signal at different time points. (F) Ex vivo imaging of the FePt microrollers. (G) Photoacoustic (PA) projection images of all the dead mice. (H) Magnetic directional drive of microrollers in blood vessels. Reproduced with permission from [147]. Copyright 2021 The Authors. Advanced Functional Materials published by Wiley-VCH GmbH.

Over the past few decades, these Janus MNMs composed of separate compartments have been extensively employed in various imaging techniques, such as optical, ultrasound, photoacoustic, and magnetic resonance imaging (MRI) [139]. Their dynamic behavior enhances imaging contrast compared to static particles [140,141]. Lin et al. [142] developed a NIR light-driven Janus nanorobot to visualize intracellular miRNAs. This nanomotor overcomes the limitation of free diffusion of probes in miRNA detection, resulting in a 1.72-fold increase in signal intensity. MRI has emerged as an effective noninvasive tool for in vivo imaging due to its ability to produce high-resolution images of deep tissues in arbitrary 3D planes [143,144]. However, the limited penetration of imaging agents in solid tumor tissues and cells poses a substantial challenge to achieving adequate visibility for cancer diagnosis. To overcome this limitation, Wang and colleagues [145] developed NIR light-driven nanoparticles based on Gd-doped Janus mSiO_2_ nanoparticles, which exhibited excellent photothermal properties and improved cell internalization and MRI ability by thermomechanical perforating tumor cell membranes (Fig. 6A). After the injection of the motile Janus mSiO_2_ nanomotors into mice, the MRI signal intensity in the tumor region increased approximately 1.73-fold compared to that of static Janus mSiO_2_ nanoparticles (Fig. 6B). Each imaging modality can be combined with other modalities to maximize their benefits [146]. Sitti and colleagues [147] developed magnetically actuated Janus surface microrollers by half-coating silica particles with ferromagnetic FePt alloy nanofilms (Fig. 6C). The newly developed FePt-coated microrollers outperform FePt-coated microrollers in magnetic properties, as the 60-nm-thick FePt film exhibits higher coercivity compared to the Ni-coated group (Fig. 6D). Additionally, the FePt-coated microrollers demonstrate superior biocompatibility, maintaining over 85% cell viability, in contrast to less than 20% for the Ni-coated group (Fig. 6E). Notably, the FePt coating enables real-time multimodal imaging using microlasers in magnetic resonance and photoacoustic modes without additional contrast agents (Fig. 6F and G). High-resolution photoacoustic imaging demonstrated the potential application of these FePt-coated microvessels for navigation control in a simulated circulatory system, revealing upstream motion capabilities at an average flow rate of 4.5 cm s^−1^ (Fig. 6H). Ma and colleagues [148] developed a novel JN combining multiple diagnostic and therapeutic functions. Upon injection into tumor-bearing mice, the nanomotor enabled clear visualization of tumor location and contours through fluorescence imaging/photoacoustic imaging, providing real-time monitoring for 808-nm NIR light-triggered PDT/PTT synergistic therapy. The active motion of the nanoparticles opens numerous possibilities for new approaches in cancer theragnostic.

### Biosensing

Biosensors are devices with recognition elements that selectively bind to specific biological substances and convert biophysical or chemical reactions into measurable signals [149–151]. The surface of Janus MNMs can be conveniently functionalized, enabling them to identify and detect target molecules, making them suitable for application as biosensors in detecting specific substances in living organisms [152]. The utilization of static Janus emulsions may hinder effective interaction with target receptors. However, the integration of self-propelled Janus sensors presents a promising solution to this limitation by enhancing receptor engagement. Moreover, fluorescence sensing emerges as a highly promising approach for the detection of cancer, offering potential for advancements in diagnostic methodologies. Zhao et al. [153] demonstrated this application by constructing Janus rods with CAT grafted onto one side of the structure to serve as a power source for motion-enhanced capturing of circulating tumor cells (CTCs). Thymine and guanine were conjugated with tetraphenylethene and fluorescein isothiocyanate to achieve radiometric fluorescence sensing and then grafted onto aptamers via base-pair interactions. These modified aptamers were subsequently tethered covalently to the other side of the Janus rods (Fig. S6A). The morphology of the JMs with an aspect ratio of 4 was shown in Fig. S6B. When presented with H_2_O_2_ fuel, the CAT-catalytic reaction propelled the Janus rods, and TLS11a aptamer provided high-affinity capture of tumor cells. Upon specific capture of CTC, the JMs transitioned from blue to green due to the release of tetraphenylethene and fluorescein isothiocyanate (Fig. S6C). Figure S6D illustrates the pronounced contrast in fluorescence emitted by the JMs following incubation with HepG2 under a UV lamp. Before treatment with HepG2, the JMs with an aspect ratio of 2 (JM-2) suspension emitted conspicuous blue fluorescence. However, the emission underwent a marked and distinct change to bright green fluorescence following exposure to HepG2. Ma and colleagues [154] developed a Janus Au@mSiO_2_@Pt motor probe capable of autonomous motion, which enhances antigen–antibody interactions’ binding probability and efficiency. By using 5 wt % H_2_O_2_ as a buffer, the Janus Au@mSiO_2_@Pt-based lateral flow immunoassays allow for quantitative detection of both PG I and PG II within 10 min, with detection limits of 2.2 and 2.1 μg ml^−1^, respectively. Russell et al. [155] presented a Janus sensor for procalcitonin detection, which consists of colored iron oxide cores and Janus coatings that exhibit both biological specificity and motion characteristics. The antibodies in the Janus coating capture the target molecule and CAT (Fig. S6E), leading to autonomous motor motion. The movement of the motor on the filter paper results in a transition from numerous colored spots to larger, fewer colored spots (Fig. S6F). The color change analysis enables the rapid detection of the sepsis biomarker calcitonin in whole blood within 13 min. The electrochemical sensor converts the electrochemical reaction into an easily interpretable signal. Wang and colleagues [156] developed Fe_3_O_4_@SiO_2_/Pt nanomotor sensors labeled with primary antibody immunoglobulin G (IgG). Core-shell Au@Ag nanocubes were used as secondary antibody IgG labels to amplify the IgG detection signal generated by Ag oxidation. Glassy carbon electrodes themselves could not generate a current signal, but when Au@Ag nanocubes were modified to the glassy carbon electrodes, a distinct current signal was observed by differential pulse voltammetry. Autonomous movement of the motor helps capture the target molecule and provides a method for simple and sensitive detection of immune proteins in biosensing. Zhang et al*.* [157] developed Janus mesoporous microsphere/Pt-based (meso-MS/Pt) nanostructures that enhance target delivery and accelerate the recognition process for miRNA amplification and detection in complex biological samples. Specifically, under identical experimental conditions, the fluorescence intensity of a solution containing 3% H_2_O_2_ increased faster and more strongly than that of a solution without H_2_O_2_. After 30 min, the fluorescence intensity of the H_2_O_2_-containing solution was 2.95 times higher than that of the solution without H_2_O_2_ (Fig. S6G to I). The constructed meso-MS/Pt/DNA motors effectively captured and detected target molecules, demonstrating their potential for sensitive and efficient miRNA detection.

## Conclusions and Outlook

Janus structure nanomotors, characterized by 2 sides with different properties or functionalities, represent a vital area of interest in nanomotor research over the past decade. These tiny devices demonstrate continuous motion that enhances mass transfer and improves reaction in drug delivery, cancer therapy, bioimaging, and biosensing. Designing artificial MNMs that can be applied practically is critical in nanotechnology. The utilization of asymmetric fields is pivotal in driving nanomotors by disrupting their static balance. Notably, JPs offer utility due to their anisotropic composition, chemistry, and surface features, enabling the seamless execution of multiple tasks.

In this review, we provide a comprehensive overview of the synthesis and locomotion of Janus MNMs inspired by various driving mechanisms and their applications in biomedicine. Despite the immense potential of these MNMs in diverse applications, critical challenges still need to be addressed, particularly in terms of their speed and directional control. The complex fabrication procedures, stringent operational conditions, limited propulsive forces, and imprecise motion trajectories hinder their development and practical utilization. The features of each type of MNMs, including the advantages and limitations, are summarized in Table S1. We can overcome current developmental barriers by gaining a profound understanding of these limitations. While remarkable progress has been made, focusing on tackling the pressing challenges ahead is imperative to facilitate further advancements in practical applications. Therefore, we highlight both the scientific and practical challenges and opportunities that this emerging field presents for future exploration and achievements.

### Controlled synthesis of MNMs with Janus structure

The motion behavior of MNMs is primarily determined by their structure, which requires a certain energy gradient to achieve directional movement. An asymmetric field around MNMs is essential for successful driving, which cannot be achieved with single-component structures. However, complex construction methods for anisotropic Janus structure limit the scale-up production of MNMs. A key challenge in the fabrication process of Janus MNMs is ensuring that both sides of the motor have different properties, such as different surface chemistries or magnetic properties. This requires careful material selection and precise control over the synthesis conditions. While several fabrication strategies have been proposed recently, each method has strengths and limitations. Noble metal deposition on one side of prefabricated particles is a common and effective method [158,159]. Still, the arrangement of particles on the substrate surface and suppression of rotational diffusion are crucial [160,161]. Alternatively, controlling the nucleation and growth of the second component on prefabricated particle surface can also result in JPs, but this method is sensitive to experimental parameters [162]. Despite some progress, current preparative strategies have limitations in large-scale production with high reproducibility. As the size of these structure increases, their responsiveness to external stimuli decreases due to long diffusion times, which affects their efficiency. More research efforts are needed to develop simple, general, robust, and reliable methods that can produce high-quality Janus MNMs with fine structure and long service life for practical applications on a large scale [163].

### Improvement of driving force for the MNMs

In chemically driven MNMs, their effective movement largely relies on high concentrations of fuel decomposition. However, the limited concentration of natural chemical fuels in vivo and the coexistence of different chemical gradients in the surroundings may have an undesirable impact on the chemotactic movement of MNMs [164,165], which limits the application of MNMs in biomedicine and other fields. Moving forward, it is urgently necessary to optimize synthetic chemotactic motors by improving the utilization efficiency of chemical fuels and expanding the effective operation range of the signal sources. One strategy to increase the utilization efficiency of chemical fuels is to enhance the sensitivity of the motors to signal sources by tailoring the surface properties of the motors to improve the motion behaviors. An alternative strategy is to introduce signal amplification systems that can sense environmental clues and transduce them into secondary signals to regulate the motion behaviors of motors. These approaches can also apply to the other types of motors that are propelled by light, electric field, and ultrasound to enhance driving force and improve motion behaviors.

### Guidance/navigation of self-propelled MNMs

Among the various potential applications proposed for MNMs, targeted cancer chemotherapy, especially for a solid tumor, has received the most attention and is a hot research topic for medical nanorobot research [166]. The concept of targeted cancer chemotherapy relies on the ability of MNMs to transport biomedical cargo specifically to cancer cells without affecting healthy cells or tissues [167]. This concept is closely related to the navigation and directional transport capability of MNMs to the tumor site. However, the navigation of nanoscale MNMs is still a complicated and challenging task, which is always hindered by some external factors such as viscosity and Brownian diffusion [168]. For the most cancer-targeted therapy, nanomotors will most likely be injected into the bloodstream. The high ionic strength (salt concentration), fluid flow of blood, and interstitial fluids pose another critical challenge to the guidance/navigation of self-propelled MNMs. Although the direction and speed of the motor can be guided by the external field (magnetic and electric) or the intrinsic tactic behavior of MNMs (chemotaxis, phototaxis, and thermotaxis), it is still challenging to precisely control its trajectory and direction in fast blood flows. Therefore, it is one of the research directions in the future to realize the precise control of the movement direction by controlling the composition and structure of the motor. One promising direction for the improved navigation of self-propelled MNMs is to incorporate artificial intelligence and machine learning techniques into their design. By training nanomotors to recognize specific targets, such as cancer cells, it may be possible to enhance their navigational abilities and improve their effectiveness in various applications. Overall, the navigation performance of nanomotors is highly dependent on their design and the environment in which they operate. With continued research and development efforts, it is expected that nanomotors will continue to exhibit exceptional navigating capabilities.

### The survival of MNMs

The stability of MNMs is critical for their successful applications in vivo. Once introduced into the bloodstream, MNMs are immediately coated by a layer of blood plasma proteins, forming what is known as a “protein corona” [169]. This process can substantially alter the surface characteristics and properties of the MNMs, which could lead to disastrous consequences for the chemically propelled MNMs, as most of the catalytic reactions occur on their surface. To overcome this challenge, it is necessary to synthesize MNMs that can withstand the biological barriers in vivo. This will increase the number of MNMs that reach their intended targets and improve the overall treatment efficiency. With the advancements in MNM systems and increased understanding of related principles, cost-effective, efficient, intelligent, and biocompatible MNMs are expected to soon revolutionize biomedical and environmental fields.

## Data Availability

Data availability does not apply to this article as no new data were created or analyzed in this study.
