# Supplementary material for: Janus-Structured Micro/Nanomotors: Self-Propelled Mechanisms and Biomedical Applications
Source: Biomater Res. 2025 Apr 5;29:0155. doi: 10.34133/bmr.0155 (PMC11971528; doi:10.34133/bmr.0155)
Supplement: Supplementary 1 — Figs. S1 to S6 Table S1 [file bmr.0155.f1.zip › Supplemental Materials-Fig.S1-S6.docx]

***Supplemental Materials***

**Janus Structured Micro/Nanomotors: Self-Propelled Mechanisms and Biomedical Applications**

Haoyan Cheng^1†^, Beng Ma^1†^, Anqi Ji^1^, Haonan Yao^1^, Pan Chen^2^, Wenyang Zhai^1^, Shegan Gao^2^, Linlin Shi^2*^ and Hao Hu^1*^

^1^School of Materials Science and Engineering, The First Affiliated Hospital, College of Clinical Medicine, Henan University of Science and Technology, Luoyang 471023, P. R. China. ^2^Henan Key Laboratory of Microbiome and Esophageal Cancer Prevention and Treatment, Henan Key Laboratory of Cancer Epigenetics, The First Affiliated Hospital, College of Clinical Medicine, Cancer Hospital, Henan University of Science and Technology, Luoyang 471023, P. R. China.

*Address correspondence to: [huhao@haust.edu.cn](mailto:huhao@haust.edu.cn) (H. Hu); [celine_shih@haust.edu.cn](mailto:celine_shih@haust.edu.cn) (L. Shi)

^†^These authors contributed equally to this work.

**
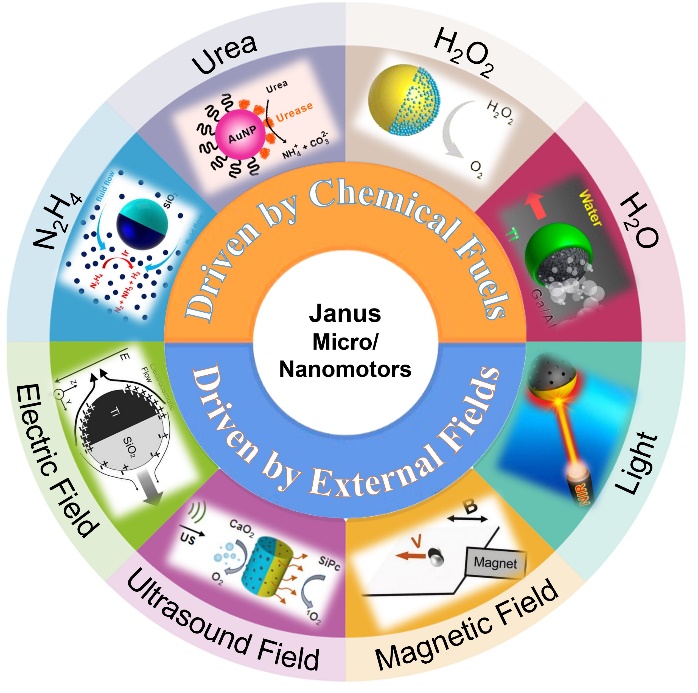
**

**Fig. S1.** Overview of MNMs driven by chemical power systems and external fields. (N_2_H_4_) Reproduced with permission from [43]. Copyright 2014 American Chemical Society. (Urea) Reproduced with permission from [52]. Copyright 2023 American Chemical Society. (H_2_O) Reproduced with permission from [45]. Copyright 2012 American Chemical Society. (H_2_O_2_) Reproduced from [7] with permission from Wiley-VCH GmbH, Copyright 2020. (Light) Reproduced with permission from [71]. Copyright 2012 American Chemical Society. (Electric field) Reproduced with permission from [114]. Copyright 2019 American Chemical Society. (Ultrasound field) Reproduced with permission from [108]. Copyright 2021 American Chemical Society. (Magnetic field) Reproduced from [104] with permission. Copyright 2018 The Authors. Published by WILEY‐VCH Verlag GmbH & Co. KGaA, Weinheim.


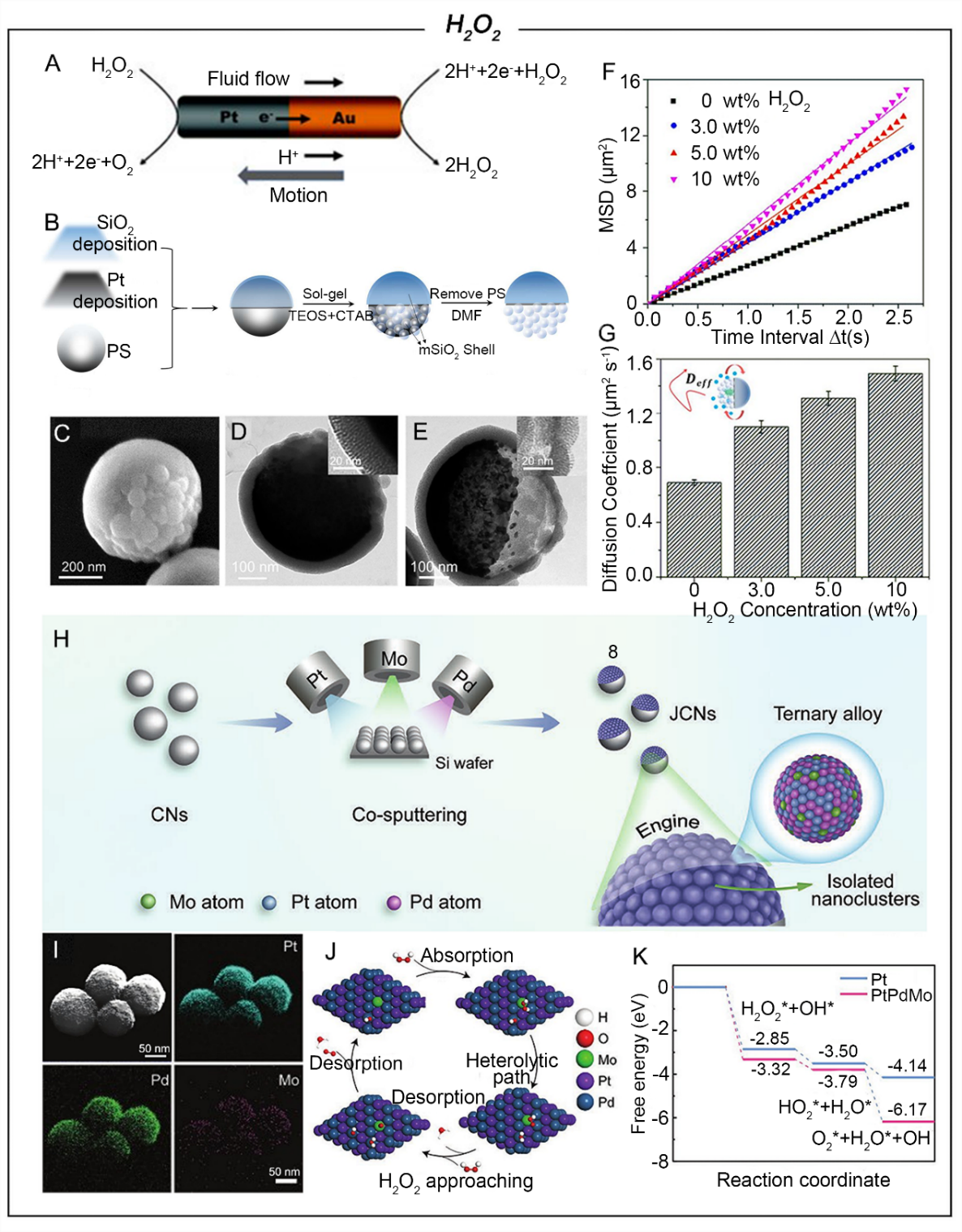


**Fig. S2.** (A) Au–Pt bimetallic nanomotor. Reproduced with permission from [30]. Copyright 2006 American Chemical Society. (B) Schematic illustration of the steps in fabricating the reversed Janus motors and the various motility scenarios. (C) Scanning electron microscope (SEM) and (D) Transmission electron microscope (TEM) images of the nanomotor before removal of the polystyrene template. (E) TEM image of the nanomotor after removal of the polystyrene template. (F-G) The effective diffusion coefficient of the nanomotors at various concentrations of H_2_O_2_. Reproduced with permission from [31]. Copyright 2016 American Chemical Society. (H) Schematic illustration of the preparation process of Janus catalysis-driven nanomotors (JCNs). (I) The corresponding element mapping of JCNs. (J) Proposed catalytic mechanism of the JCNs. (K) The calculated absorption energy and reaction energy profiles for H_2_O_2_ decomposition on the JCNs surface (red) and Pt-JCNs surface (blue) in an essential medium. Reproduced from [32] with permission from Wiley-VCH GmbH, Copyright 2022.

**Note**

***i)*** Bubble-driven MNMs typically require high concentrations of fuel, and thus, the driving speed can be controlled by adjusting the fuel concentration. However, this type of MNM has a relatively low energy efficiency (10^-9^-10^-10^), as propulsion only occurs during the moment of bubble ejection [118]. Additionally, bubble propulsion-based MNMs exhibit high propulsion rates, enhanced fluid mixing, and improved mass transfer performance, making them highly promising for applications in sensing and environmental remediation [118].

***ii)*** Phoresis-driven MNMs constructed from materials such as Pt-Au operate primarily by inducing asymmetric redox reactions on their surface, leading to an asymmetric distribution of reaction products around the MNM. If the products are charged, an electric field can be generated to propel the MNM [30]. Compared to bubble propulsion, MNMs based on the self-electrophoretic mechanism exhibit significantly higher propulsion efficiency (10^-6^-10^-9^). However, the construction of the electric field is highly challenging, and it is prone to failure in high ionic strength environments, which significantly limits the application of self-electrophoretic-driven MNMs in various fields [118].


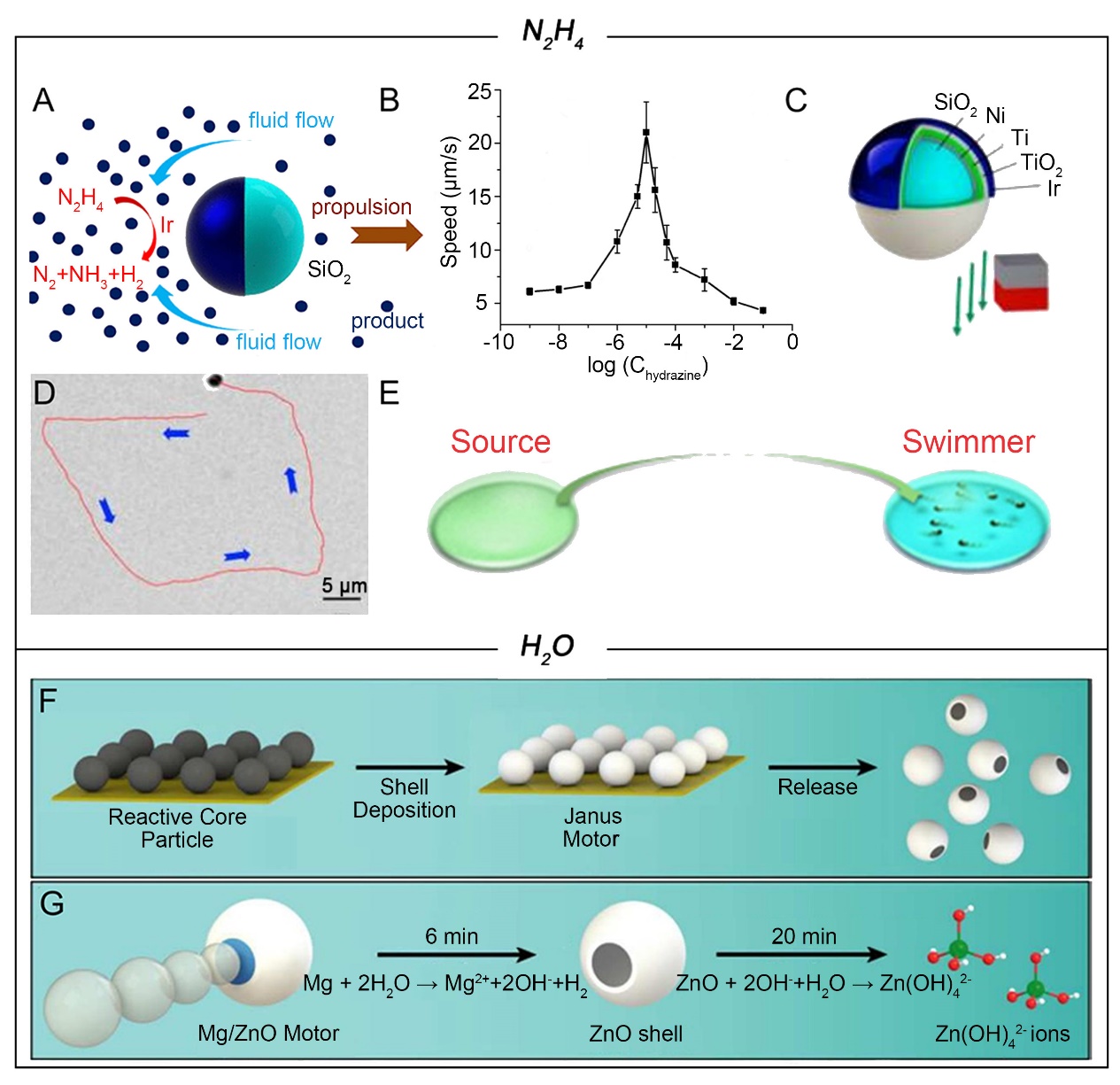


**Fig. S3.** (A) Schematic of catalytic Ir/SiO_2_ JMs powered by N_2_H_4_. N_2_, H_2_, and NH_3_ molecules are generated at the Ir surface. (B) Dependence of the speed of Ir/SiO_2_ JMs upon the N_2_H_4_ concentration over the 0.0000001-10% range. (C) Schematic of the magnetic control of multilayer Ir-TiO_2_-Ni-Ti-SiO_2_ JMs. (D) Time-lapse images showing the magnetically guided propulsion of an Ir-TiO_2_-Ni-Ti-SiO_2_ micromotor. Reproduced with permission from [43]. Copyright 2014 American Chemical Society. (E) Propulsion of Ir-Au micromotors by external N_2_H_4_ diffusing from the fuel source droplet through the surrounding atmosphere into the motor droplet. Reproduced under the terms of the Creative Commons Attribution 4.0 International License.[44] Copyright 2015, Springer Nature. (F) The fabrication of JMs. (G) The design of Mg/ZnO JMs. Reproduced with permission from [46]. Copyright 2016 American Chemical Society.


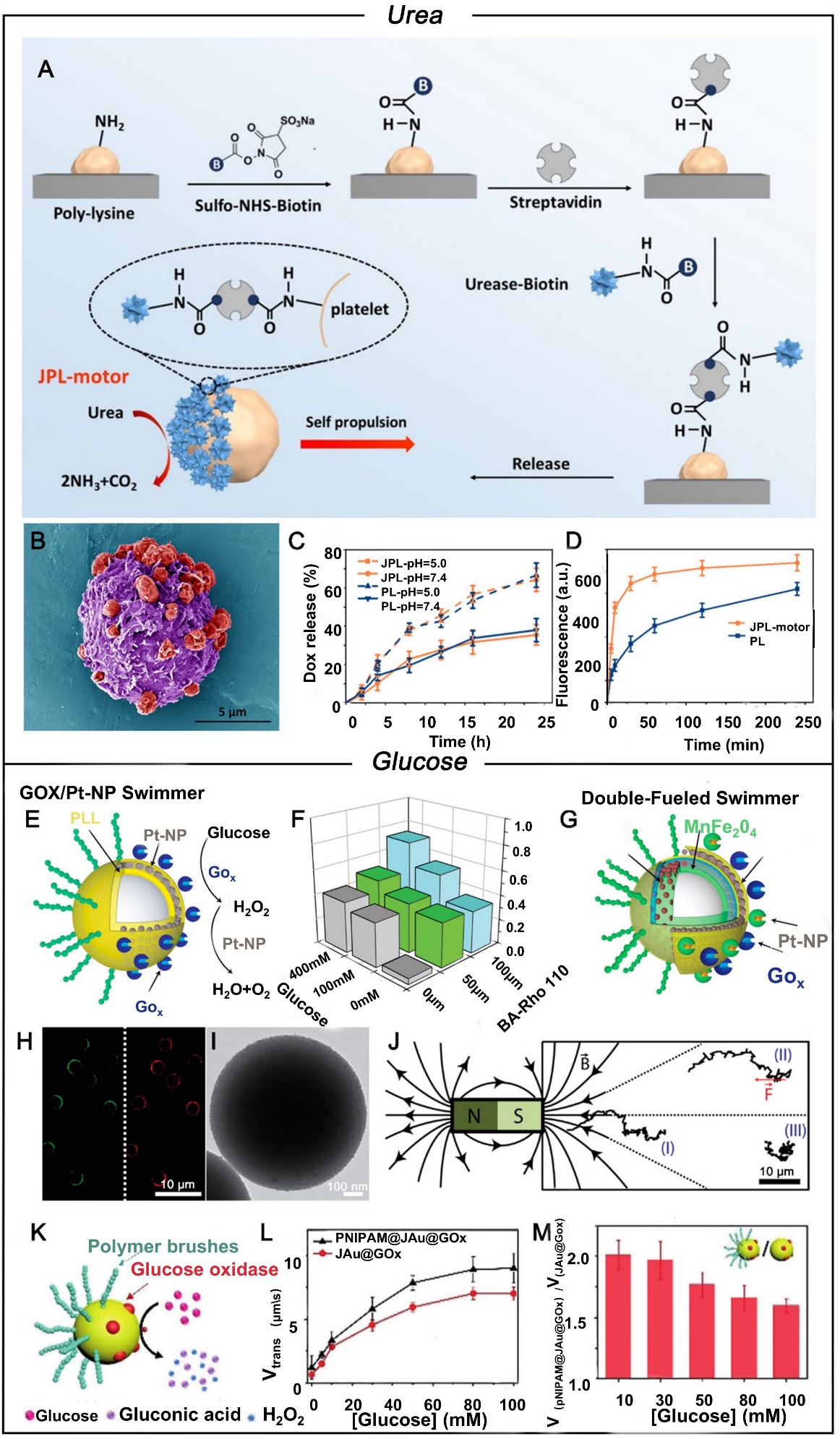


**Fig. S4.** (A) Schematic of the fabrication of JPL motors. (B) Pseudocolored SEM image of numerous JPL motors (red) attached to an MDA-MB-231 cell (purple). (C) Cumulative release from JPL-motors and unmodified platelets. (D) Comparison of the fluorescence intensity of cells after incubation with DiD-labeled JPL-motors or unmodified platelets. Reproduced with permission from [56]. Copyright 2020 The American Association for the Advancement of Science. (E) Schematic representation of the Janus-shaped GOx/Pt-NP swimmers. (F) The velocity of doubled-fueled swimmers is a function of the different fuel mixtures. (G) Double-fueled Janus swimmer with magnetostatic behavior. (H) Representative images showing the co-deposition. (I) Representative TEM image of the swimmers. (J) Representative trajectories of the double-fueled swimmers. Reproduced with permission from [9]. Copyright 2017 American Chemical Society. (K) Scheme of the motion of PNIPAM@JAu@GOx nanoswimmers in glucose solutions. (L) Dependence of the velocity of PNIPAM@JAu@GOx and JAu@GOx nanoswimmers upon glucose concentration. (M) The ratio of velocity of PNIPAM@JAu@GOx nanoswimmers to the velocity of JAu@GOx nanoswimmers. Reproduced with permission from [65]. Copyright 2019 Wiley‐VCH Verlag GmbH & Co. KGaA, Weinheim.

^
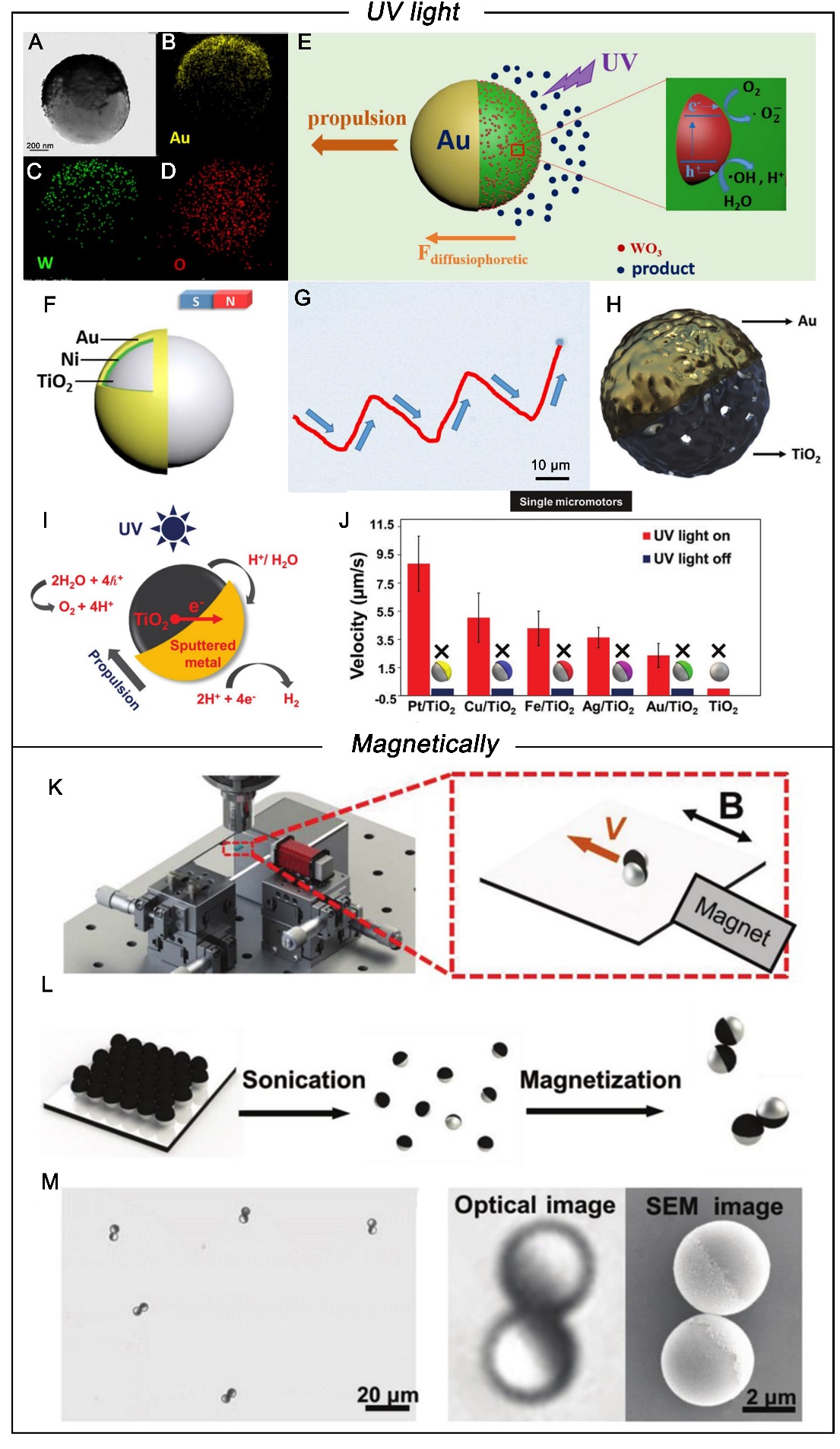
^

**Fig. S5.** (A-D) TEM image and corresponding EDX mapping images for Au, W and O of Au-WO_3_@C JMs. (E) Schematic of the driven mechanism of UV light-driven Au-WO_3_@C sphere JMs. Reproduced with permission from [90]. Copyright 2017 American Chemical Society. (F) Schematic structure of Au-Ni-TiO_2_ JMs. (G) The magnetically guided propulsion of an Au-Ni-TiO_2_ micromotor under UV light. Reproduced with permission from [91]. Copyright 2016 American Chemical Society. (H) JHP-TiO_2_-Au micromotor. Reproduced with permission from [93]. Copyright 2018 WILEY‐VCH Verlag GmbH & Co. KGaA, Weinheim. (I) The propulsion of the fabricated micromotors. (J) The average velocity of various metal/TiO_2_ micromotors in the presence (red bars) and absence (blue bars) of UV light irradiation. Reproduced with permission from [92]. Copyright 2020 WILEY‐VCH Verlag GmbH & Co. KGaA, Weinheim. Design and fabrication of microfilmer surface walkers. (K) Schematic of the experimental setup. (L) Fabrication of the microdimers. (M) Optical microscopy image of a few representative microdimers after magnetization. (N) Magnified optical microscopy image and the corresponding SEM image of a microdimer. Reproduced with permission from [104]. Copyright 2018 WILEY‐VCH Verlag GmbH & Co. KGaA, Weinheim.

**
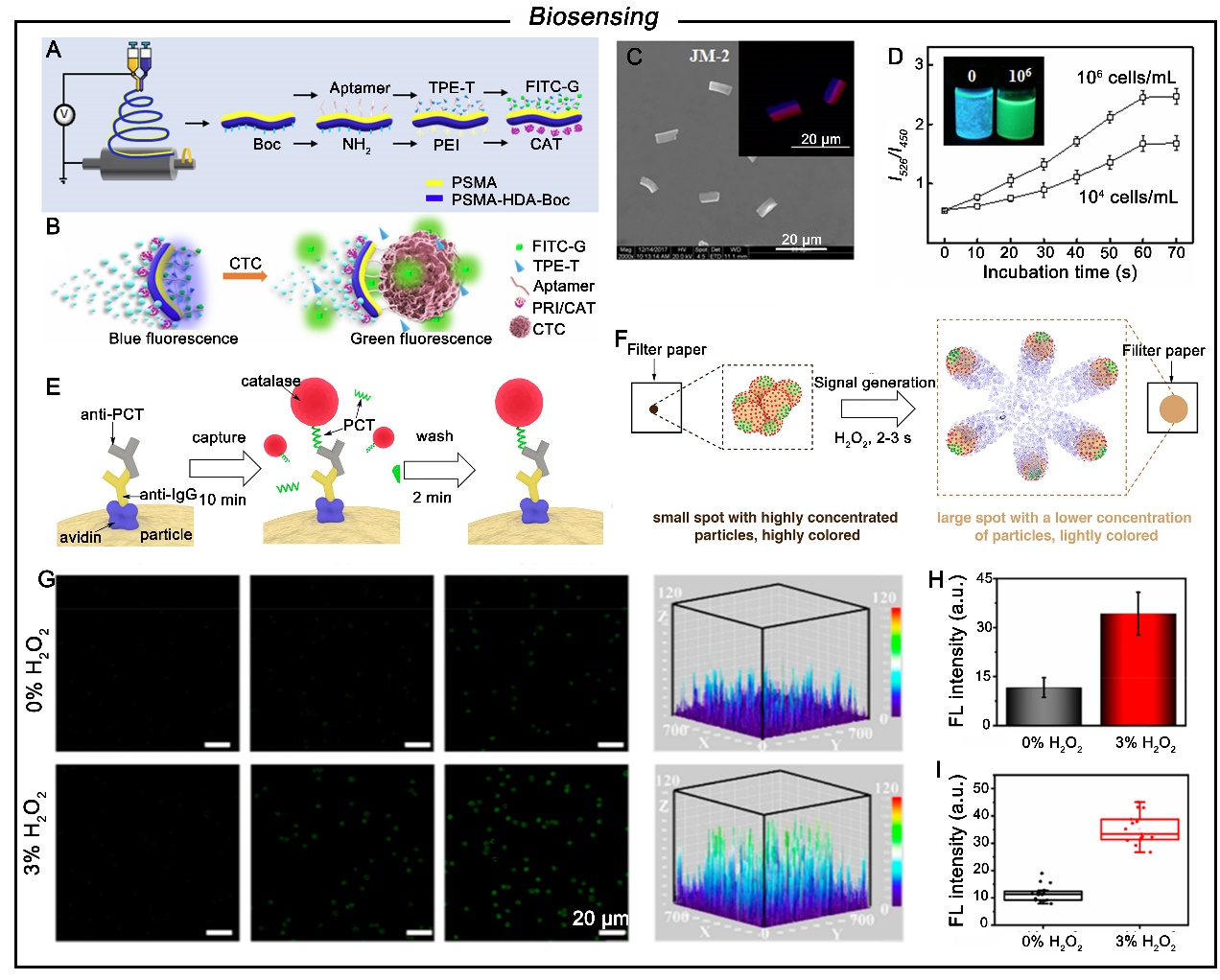
**

**Fig. S6.** (A) Preparation of self-propelled JMs. (B) The morphology of the JMs with the aspect ratios of 4. (C) Radiometric fluorescence response of JMs after the capture of CTC. (D) I526/I450 values of JM-2 suspensions after incubation for 70 s with HepG2 at 10^4^ and 10^6^ cells mL^-1^. Reproduced with permission from [153]. Copyright 2019 Elsevier B.V. All rights reserved. (E) Procalcitonin is specifically captured in blood using a competitive immunoassay. (F) Schematic representation of signal generation mechanism. Reproduced with permission from [155]. Copyright 2019 Elsevier B.V. All rights reserved. (G) Confocal Laser Scanning Microscope images of meso-MS/Pt/DNA in solution containing target and fuel probes with or without 3% H_2_O_2_ at different times. (H-I) FL intensity of (G) at 30 min. Reproduced with permission from [157]. Copyright 2022 American Chemical Society.
