# Supplementary material for: Janus-Structured Micro/Nanomotors: Self-Propelled Mechanisms and Biomedical Applications
Source: Biomater Res. 2025 Apr 5;29:0155. doi: 10.34133/bmr.0155 (PMC11971528; doi:10.34133/bmr.0155)
Supplement: Supplementary 1 — Figs. S1 to S6 Table S1 [file bmr.0155.f1.zip › Supplemental Materials-Table S1.docx]

***Supplemental Materials***

**Janus Structured Micro/Nanomotors: Self-Propelled Mechanisms and Biomedical Applications**

Haoyan Cheng^1†^, Beng Ma^1†^, Anqi Ji^1^, Haonan Yao^1^, Pan Chen^2^, Wenyang Zhai^1^, Shegan Gao^2^, Linlin Shi^2*^ and Hao Hu^1*^

^1^School of Materials Science and Engineering, The First Affiliated Hospital, College of Clinical Medicine, Henan University of Science and Technology, Luoyang 471023, P. R. China. ^2^Henan Key Laboratory of Microbiome and Esophageal Cancer Prevention and Treatment, Henan Key Laboratory of Cancer Epigenetics, The First Affiliated Hospital, College of Clinical Medicine, Cancer Hospital, Henan University of Science and Technology, Luoyang 471023, P. R. China.

*Address correspondence to: [huhao@haust.edu.cn](mailto:huhao@haust.edu.cn) (H. Hu); [celine_shih@haust.edu.cn](mailto:celine_shih@haust.edu.cn) (L. Shi)

^†^These authors contributed equally to this work.

| **Table S1.** A brief comparison of typical MNMs. | | | |  |
| --- | --- | --- | --- | --- |
| Power sources | Propulsion mechanism | Advantages | Limitations | Applications |
| H_2_O_2_ | Bubble propulsion [18-20, 31, 32, 36] | Simple and effective fuel source, high propulsion efficiency, versatility and ease of integration,  no need of additional equipment for propulsion. | Non-biocompatible chemical fuels, fuel shortage in practical application, sensitivity of self-electrophoretic propulsion to ion strength, difficulty in controlling motion. | Drug delivery  [18,19, 21, 32]  Environmental remediation [20, 39]  Gas sensing [43] |
|  | Self-diffusiophoresis [21, 30, 39, 40] |  |  |  |
|  | Self-electrophoretic [30] |  |  |  |
| N_2_H_4_ | Bubble propulsion [43, 44] |  |  |  |
| H_2_O | Bubble propulsion [45-47] | Environmentally compatible nontoxic response, use of biodegradable materials, biocompatible fuels and catalysts, chemotactic motion,  high diversity of enzyme types. | Relatively short lifetime,  limited fuel supply,  difficulty in controlling motion,  limited enzyme activity. | Drug delivery  [9, 47, 52-54, 56, 65] |
| Urea | Self-diffusiophoresis [52-54, 56] |  |  |  |
| Glucose | Self-diffusiophoresis/magnetic [9] |  |  |  |
|  | Self-diffusiophoresis/  Bubble propulsion [65] |  |  |  |
| NIR | Photothermal propulsion  [71, 75, 76] | Fuel free, long lifetime, biocompatible, highly spatial and temporal resolutions, fast response,  easy operation,  non-toxic and non-invasive,  remote activation. | Insufficient motion energy,  requirement of manipulating equipment, safety concerns of UV light, limited penetration capability of light. | Drug delivery [75, 77, 94]  Environmental remediation [80, 87] |
|  | Temperature-sensitive polymer [77] |  |  |  |
| Visible Light | Self-electrophoresis [79-81, 84, 87] |  |  |  |
| UV | Diffusiophoretic [90] |  |  |  |
|  | Self-electrophoresis [94] |  |  |  |
| Magnetically | Rotating magnetic fields [95, 103] | Reusable, long lifetime, fuel free,  biocompatible,  reversible and wireless manipulation, highly spatial and temporal resolutions, fast response,  strong penetrating ability. | Non-biocompatible and non-degradable materials，  tedious and high-cost preparation processes, requirement for specialized equipment. | Drug delivery [103]  Imaging [96, 116] |
|  | Oscillating magnetic fields [96] |  |  |  |
| Ultrasound | Standing acoustic field  [106, 108, 109] |  |  |  |
| Electrically | A. C. electric fields [113, 114, 116] |  |  |  |
|  | Dielectric fluid [115] |  |  |  |
